# Haplotype‐Resolved Genotyping and Association Analysis of 1,020 β‐Thalassemia Patients by Targeted Long‐Read Sequencing

**DOI:** 10.1002/advs.202410992

**Published:** 2024-12-31

**Authors:** Yuhua Ye, Chao Niu, Aiping Mao, Lang Qin, Jiahan Zhan, Weijie Chen, Zhentian Liu, Tiantian Xie, Qianqian Zhang, Jiaqi Li, Li Huang, Wanli Meng, Yumeng Liu, Liuhua Liao, Junqin Cai, Riyang Liu, Xinhua Zhang, Lihong Zeng, Yaoyun Li, Bin Lin, Kui Li, Xiaoyun Hua, Binbin Huang, Honggui Qin, Yueyan Huang, Zhijing Huang, Jinquan Lao, Xiang Qu, Juanjuan Chen, Xiaoqin Feng, Qiujun Liu, Wanying Lin, Xiaoman Zhou, Yidan Liang, Xingjiang Long, Jiaofeng Qin, Lixiang Yan, Weijian Zhu, Lian Yu, Chengwu Fan, Deguo Tang, Tianyu Zhong, Jufang Tan, Zhilin Ren, Xiangmin Xu

**Affiliations:** ^1^ Innovation Center for Diagnostics and Treatment of Thalassemia Nanfang Hospital Southern Medical University Guangzhou Guangdong 510515 China; ^2^ Department of Medical Genetics School of Basic Medical Sciences Southern Medical University Guangzhou Guangdong 510515 China; ^3^ Department of Long‐Read Sequencing Research and Development Berry Genomics Corporation Beijing 102200 China; ^4^ Department of Pediatrics Huizhou Central People's Hospital Huizhou Guangdong 516001 China; ^5^ Department of Hematology 923(rd) Hospital of the People's Liberation Army Nanning Guangxi 530021 China; ^6^ Department of Pediatrics 923(rd) Hospital of the People's Liberation Army Nanning Guangxi 530021 China; ^7^ Guangzhou Huayinkang Healthcare Group Co., Ltd. Guangzhou Guangdong 510663 China; ^8^ Department of Internal Medicine Sixth People's Hospital of Nanning Nanning Guangxi 530022 China; ^9^ Department of Pediatrics Affiliated Hospital of Youjiang Medical University for Nationalities Baise Guangxi 533000 China; ^10^ Department of Pediatrics Liuzhou Worker's Hospital Liuzhou Guangxi 545005 China; ^11^ Department of Pediatrics The Second People's Hospital of Shenzhen The First Affiliated Hospital of Shenzhen University Shenzhen Guangdong 518035 China; ^12^ Department of Pediatrics Nanfang Hospital Southern Medical University Guangzhou Guangdong 510515 China; ^13^ Department of Laboratory Medicine Nanfang Hospital Southern Medical University Guangzhou Guangdong 510515 China; ^14^ Central Laboratory Dongguan Songshan Lake Central Hospital Dongguan Guangdong 523808 China; ^15^ Department of Pediatrics Liuzhou People's Hospital Liuzhou Guangxi 545001 China; ^16^ Medical Genetics Laboratory Heyuan Maternal and Child Health Care Hospital Heyuan Guangdong 517000 China; ^17^ Department of Hematology and Oncology Zhuhai People's Hospital The Third Affiliated Hosptial Jinan University Medical College Zhuhai Guangdong 519000 China; ^18^ Department of Hematology and Rheumatology Longyan First Hospital Affiliated to Fujian Medical University Longyan Fujian 364000 China; ^19^ Department of Pediatrics Second people's hospital of Guilin Guilin Guangxi 541001 China; ^20^ Department of Genetics and Laboratory Medicine Maternal and Child Health Hospital of Yongzhou City Yongzhou Hunan 425000 China; ^21^ Department of Clinical Laboratory The First Affiliated Hospital of Gannan Medical University Ganzhou Jiangxi 341000 China; ^22^ Prenatal Diagnosis Center Chenzhou First People's Hospital Chenzhou Hunan 423000 China

**Keywords:** fetal hemoglobin, thalassemia, third‐generation sequencing

## Abstract

Despite the well‐documented mutation spectra of β‐thalassemia, the genetic variants and haplotypes of globin gene clusters modulating its clinical heterogeneity remain incompletely illustrated. Here, a targeted long‐read sequencing (T‐LRS) is demonstrated to capture 20 genes/loci in 1,020 β‐thalassemia patients. This panel permits not only identification of thalassemia mutations at 100% of sensitivity and specificity, but also detection of rare structural variants (SVs) and single nucleotide variants (SNVs) in modifier genes/loci. The highly homologous regions of α‐/β‐globin gene clusters are then phased and 3 novel haplotypes in *HBG1/HBG2* region are reported in this population of β‐thalassemia patients. Furthermore, one of the haplotypes is associated with ameliorated symptoms of β‐thalassemia. Similarly, 5 major haplotypes are identified in *HBA1/HBA2* homologous region while one of them is found highly linked with deletional α‐thalassemia mutations. Finally, rare mutations in erythroid transcription factors in *DNMT1* and *KLF1* associated with increased expression of fetal hemoglobin and reduced transfusion dependencies are identified. This study presents the largest T‐LRS study for β‐thalassemia patients to date, facilitating precise clinical diagnosis and haplotype phasing of globin gene clusters.

## Graphical abstract

A targeted long‐read sequencing (T‐LRS) panel for accurate genotyping of β‐hemoglobinopathies is developed and its performance is tested in 1,020 β‐thalassemia patients. Novel haplotypes in the globin gene clusters are detected and functional rare variants are found by association analysis. This study presents the largest T‐LRS study for β‐thalassemia patients to date, facilitating precise clinical diagnosis and haplotype phasing of globin gene clusters.

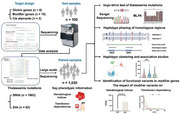

## 1 Introduction

Thalassemia is one of the most common monogenic disorders affecting 4.4 out of every 10 000 live births worldwide.^[^ ^1^ ^]^ The two clinical forms, α‐ and β‐thalassemia are characterized by imbalanced synthesis of α‐ and β‐globin due to the genetic defects majorly in *HBA1/HBA2* and *HBB*,^[^ ^2^ ^]^ which trigger the pathogenesis of ineffective erythropoiesis, increased hemolysis, and iron overload.^[^ ^3^, ^4^, ^5^ ^]^ Patients with severe forms of β‐thalassemia mostly require life‐long blood transfusion though emerging cases have been reported cured by allogeneic stem cell transplantation or gene‐editing of hematological stem cells.^[^ ^6^ ^]^ Population screening and molecular diagnosis is thus considered as an effective strategy for the prevention of the birth defect.

Traditional routine strategy for the screening of thalassemia carriers relies primarily on the thalassemic traits as evaluated by their hematological indices and hemoglobin components prior to molecular diagnosis.^[^ ^7^, ^8^ ^]^ However, limitations also arise regarding its time‐effectiveness and the potential risk of missed detections in cases with “silent” or “rare” mutations or complex structural variants.^[^ ^7^, ^9^ ^]^ To tackle these challenges, significant efforts have been dedicated to developing a high‐throughput system for the comprehensive detection of thalassemia mutations using next‐generation sequencing (NGS) and long‐read sequencing (LRS), independent of prior knowledge of hematological indices.^[^ ^10^, ^11^, ^12^, ^13^ ^]^ Up to date, haplotype phasing of these genomic regions involving the disease‐causing mutations of thalassemia is mainly based on linkage disequilibrium (LD) test among the population of thalassemia patients or carriers.^[^ ^14^, ^15^ ^]^ Apart from detecting disease‐causing mutations of thalassemia, comprehensive analysis of modifier variants in β‐thalassemia patients might advance precise diagnosis and genetic therapies for treating these monogenic diseases.^[^ ^16^ ^]^

To date, robust evidence from population‐based studies has shown a wide range of clinical severity in β‐thalassemia patients, even among those with identical disease‐causing mutations.^[^ ^2^, ^17^ ^]^ This enabled population‐based genome‐wide association studies and the identification of key modulator genes and loci, including *BCL11A*, *KLF1*, *GATA1*, *MYB‐HBS1L*, and *HBG* promoters associated with the levels of fetal hemoglobin (HbF).^[^ ^18^, ^19^, ^20^, ^21^ ^]^ Moreover, in vitro validations have led to the identification of key regulatory role of trans‐acting complexes and members on hemoglobin switching.^[^ ^22^, ^23^, ^24^ ^]^ Some of these targets, such as the erythroid‐specific enhancer in *BCL11A* and promoters of *HBG* genes, were applied as targets for gene‐editing of autologous stem cells as a genetic therapy of β‐thalassemia and sickle cell anemia.^[^ ^25^, ^26^, ^27^, ^28^ ^]^ These findings highlighted the critical role of population‐based screening of naturally occurring variants modifying the clinical symptoms of thalassemia patients. We previously developed a T‐LRS panel with the PacBio Sequel II platform to identify single‐nucleotide variants (SNVs) and structural variants (SVs) within long‐range fragments, designed as targeted amplicons covering all known regions associated with disease‐causing mutations in thalassemia. This approach, termed Comprehensive Analysis of Thalassemia Alleles (CATSA), has demonstrated its advantages in precise prenatal diagnosis and genetic counseling compared to traditional PCR‐based methods.^[^ ^29^, ^30^ ^]^ In this study, we optimized the panel by additionally capturing all cis‐elements in globin gene clusters and modifier genes known to regulate hemoglobin switching. We next introduced a cohort of 1020 β‐thalassemia patients to perform T‐LRS with this panel to test its performance in the detection of thalassemia genotypes. Furthermore, the major haplotypes in the highly homologous *HBG1/HBG2* and *HBA1/HBA2* regions were characterized using the long‐sequencing reads, followed by haplotype‐based association studies. Finally, we provided the mutation spectra among the modifier genes captured in this panel and uncovered a total of 198 variants associated with the expression of HbF in the 1020 β‐thalassemia patients. Taken together, our study provided the general phasing profiles and spectra of modifier variants in a large cohort of β‐thalassemia patients through application of an optimized T‐LRS panel.

## 2 Experimental Section

### 2.1 The Design and Experimental Procedures of the LRS Panel

**Figure 1:**
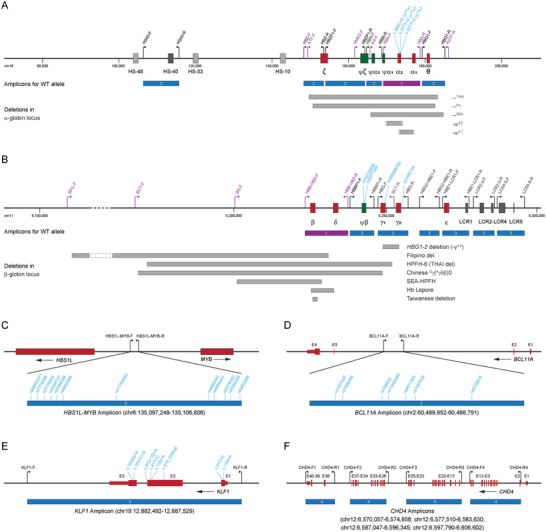
Design of the PCR primers for the T‐LRS panel. A,B) Primers designed in the α‐globin (A) and β‐globin (B) gene loci. The purple primers were included in reaction 1 (upgraded CATSA assay) to cover SNVs/indels in *HBA1*, *HBA2*, *HBB*, and *HBD* genes, as well as common deletions in the gene loci. The black primers were designed to cover other core genes and regulatory elements in the α‐globin (A) and β‐globin (B) gene loci. C) Primer pair included in reaction 3 for the *HBS1L*‐*MYB* amplicon to cover *HBS1L*‐*MYB* intergenic polymorphism. D) Primer pair included in reaction 2 for the *BCL11A* amplicon located in the intron 2 of *BCL11A*. E) Primer pair included in reaction 3 for the *KLF1* amplicon to cover the full‐length gene. F) Four primer pairs included in reaction 4 for the four *CHD4* amplicons to cover the core exons and the majority of introns for *CHD4*.

The T‐LRS panel contained four long‐range multiplex PCR reactions to target known disease‐causing variants of thalassemia, core regulatory elements in α‐ and β‐globin gene loci, known and potential novel modifier genes of β‐hemoglobinopathies (**Figure** **1**A–F; Figure S1 and Table S1, Supporting Information). Specifically, reaction 1 was an upgraded LRS approach modified from previously published CATSA assay,^[^ ^31^ ^]^ to incorporate new primers for the detection of *HBD* gene and large deletions in the β‐globin locus (Figure 1A,B). Reaction 2 contained 10 pairs of primers to target other core genes and regulatory elements in the α‐globin locus, and five modifier genes including *BCL11A, GATAD2A, DNMT1, GATA1*, and *ZBTB7A* (Figure 1A,D; Figure S1, Supporting Information). Reaction 3 contained eight pairs of primers to target other core genes and regulatory elements in the β‐globin locus, and two modifier genes including *HBS1L‐MYB* and *KLF1* (Figure 1B,C,E). Reaction 4 contained 11 pairs of primers to target four potential modifier genes including *CHD4, KLF3, KLF8*, and *SIRT1* (Figure 1F; Figure S1, Supporting Information). To achieve accurate genotyping of thalassemia alleles and modifier variants in β‐hemoglobinopathies, primers for multiplex LR‐PCR for a T‐LRS gene panel were designed to cover known disease‐causing variants in the α‐/β‐globin gene clusters and modifier genes/loci mentioned above (Figure 1; Figure S1, Supporting Information). Vigorous tests of primer sequences and concentration were performed for all the four reactions to achieve optimized balanced amplification among fragments (Figure S2, Supporting Information).

PCR amplification and long‐read library preparation were performed similarly as previously described.^[^ ^31^, ^32^, ^33^, ^34^ ^]^ Multiplex long‐range PCR (LR‐PCR) was performed in 50‐µL reactions containing 10 to 100 ng of genomic DNA, 1xPCR buffer for KOD FX Neo, 0.4 mm of each dNTP, 1 µm of primer mixture, and 1 µL of KOD FX Neo (TOYOBO). PCR cycling conditions for optimal fragment amplification were 94 °C for 5 min (1 cycle), 98 °C for 10 s, and 68 °C for 12 min (30 cycles), and 68 °C for 10 min (1 cycle). Barcoded adaptors were ligated to the PCR products to construct individual sequencing library. Then each library was quantified and pooled together by equal molarity. After purification and quantification, the pooled library was converted to single‐molecule real‐time dumbbell (SMRTbell) library with Binding kit 3.2 and cleanup beads (Pacific Biosciences) and sequenced for 30 h on the Sequel II platform (Pacific Biosciences) under circular consensus sequencing (CCS) mode.

### 2.2 Sample Recruitment

A total of 1020 β‐thalassemia patients were recruited from five provinces (Guangxi, Guangdong, Fujian, Jiangxi, and Hunan) in southern China from 2019 to 2022. Patients under 3 years of age and pregnant women were excluded. Basic information including age, sex, body weight, and height of each patient were recorded. By reviewing the electronic medical records of the patients, the medical history including the age at first transfusion, blood transfusion history was collected. Pre‐transfusion peripheral blood test was conducted to obtain the hematological indices of each patient. β‐Thalassemia genotypes were primarily determined by traditional PCR‐based methods, listed in Table S1 (Supporting Information). This study was approved by the ethics committee of Nanfang Hospital, Southern Medical University, and the ethics committees at each local hospital (Approval ID: NFEC‐2019‐039). All subjects and/or their guardians provided signed informed consent for participation and this study adhered to the Declaration of Helsinki.

### 2.3 Sequencing Data Processing and Association Analysis

A customized bioinformatics pipeline was developed and optimized for detection of structure variations and SNVs/indels, construction of haplotypes for each fragment, and correlation between variants/haplotypes and phenotype of β‐hemoglobinopathies (Figures S3, S4, Supporting Information). The raw subreads in the sequencing bam files were processed to high‐fidelity CCS reads, divided by unique barcodes for different samples, and aligned to genome build hg38 in the SMRTlink software suite (Pacific Biosciences). For all the CCS reads from the four reactions, structural variations were called based on the utilization of primers on each end and the read length of CCS reads using blastn.^[^ ^35^ ^]^ SNVs and indels of all the targeted reads were called by FreeBayes 1.3.4 with read depth ≥20 (Biomatters). The pathogenicity of variants identified in α‐ and β‐globin loci was interpreted according to the general guidelines and from information provided in hemoglobin variant databases.^[^ ^36^, ^37^, ^38^ ^]^ For all the variants identified from the t‐LRS assay, the correlation between each variant and phenotype of β‐hemoglobinopathies was analyzed with Plink 1.9. For each of the 31 fragments from T‐LRS, the structural variants and variant calling files from FreeBayes were combined to generate individual variant matrix, which was then subjected to haplotype phasing using WhatsHap.^[^ ^39^ ^]^ The haplotypes of all the samples for specific fragments were clustered and displayed with ggplot2. To generally visualize the major haplotypes in all patients of the cohort, the alleles of each locus were marked as either 0 or 1 to represent wild type or mutant allele in each patient and formed a matrix. The matrix was then deduplicated by row and clustered using pheatmap (v1.0.12) package with Pearson correlation and complete linkage method. The genotype‐phenotype association studies were conducted by generalized linear model method and visualized using the R software package ggstatsplot (v0.12.2). Survival curves of haplogroups were calculated using the Kaplan–Meier method and compared using the log‐rank test to evaluate the impact of candidate variants on clinical symptoms of β‐thalassemia patients.

For phylogenetic analysis, the haplotypes were aligned using the MAFFT (v7.520) program with default parameters, and a phylogenetic tree was generated using FastTree (v2.1.11). The resulting tree was visualized using FigTree (v1.4.4). Haplogroups were identified using a heatmap and classified using hierarchical clustering, with different colors for distinguishment. LD and haplotype blocks were constructed using the LDBlockShow software (V1.40). Transcription factor binding sites were identified by SnapGene Viewer based on the motifs of five key erythroid regulators: *KLF1, BCL11A, GATA1, NFY*, and *TAL1*. The variants potentially altered the TFBS on the globin gene clusters were plotted using the package ggplot2.

### 2.4 Homology Modeling

To evaluate the potential effects of the missense mutations in *KLF1* and *DNMT1* on protein structures, the homology models of the wild‐type and polypeptide chains were constructed using SWISS MODEL Server.^[^ ^40^ ^]^ The one with the highest Global Model Quality Estimation (GMQE) values were selected as the optimal model for further analysis. The models were displayed using the PyMOL Molecular Graphics System, Version 1.3, Schrödinger, LLC.

### 2.5 Statistical Analysis

The false discovery rate (FDR) to reflect the statistical significance of genetic variants called by T‐LRS in the phenotype‐genotype association analysis was determined by Benjamini‐Hochberg FDR correction for multiple testing, implemented by Plink 1.9. The impact of rare mutations identified in *KLF1* and *DNMT1* on the clinical symptoms and hematological indices was evaluated by a one‐sample t‐test while the inter‐group comparisons of 1020 β‐thalassemia patients with different haplotypes in the *HBG1/2* and *HBA1/2* fragments were implemented by unpaired t‐test.

## 3 Results

### 3.1 Establishing and Optimizing a System for a T‐LRS Gene Panel

**Figure 2:**
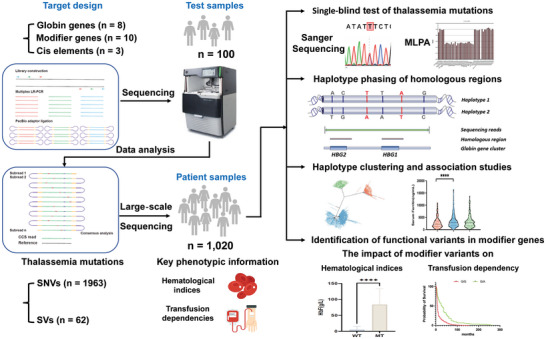
The overall flowchart of this study. The target region is composed of 8 globin genes (*HBA1*, *HBA2*, *HBB*, *HBD*, *HBG1*, *HBG2*, *HBZ*, *HBE*), 10 modifier genes (*BCL11A*, *KLF1*, *GATA1*, *GATAD2A*, *ZBTB7A*, *DNMT1*, *CHD4*, *KLF3*, *KLF8*, and *SIRT1*) and 3 cis‐elements (HS‐40 in α‐globin gene cluster, LCR in β‐globin gene cluster and the intergenic polymorphisms in *MYB‐HBS1L*). 100 samples with pre‐typed genotypes of thalassemia were recruited to test this panel with their detailed genotypes presented in Supplemental File S3. 1020 β‐thalassemia patients were then recruited for the evaluation of capability performance of this panel, from which the long‐sequencing read data were applied for variant detection, haplotypic phasing, and association studies.

This panel is designed to capture 8 globin genes (*HBA1*, *HBA2*, *HBB*, *HBD*, *HBG1*, *HBG2*, *HBZ*, *HBE*), 10 modifier genes (*BCL11A*, *KLF1*, *GATA1*, *GATAD2A*, *ZBTB7A*, *DNMT1*, *CHD4*, *KLF3*, *KLF8*, and *SIRT1*) and 3 cis‐elements (HS‐40 in α‐globin gene cluster, LCR in β‐globin gene cluster and the intergenic polymorphisms in *MYB‐HBS1L*). We then tested the practicability of this panel by recruiting 100 samples with known genotypes of thalassemia. Upon optimization of the panel, a cohort of 1020 β‐thalassemia patients were subjected to large‐scale T‐LRS, in which single‐blind test of thalassemia mutations were performed to evaluate the capability performance, followed by detection of modifier variants, haplotypic phasing, and association studies to report variants or haplotypes associated with phenotypic changes in β‐thalassemia patients. The flowchart of this study described above is shown in **Figure** **2**.

### 3.2 Detection of Disease‐Causing Variants of Thalassemia and Modifier Variants

**Figure 3:**
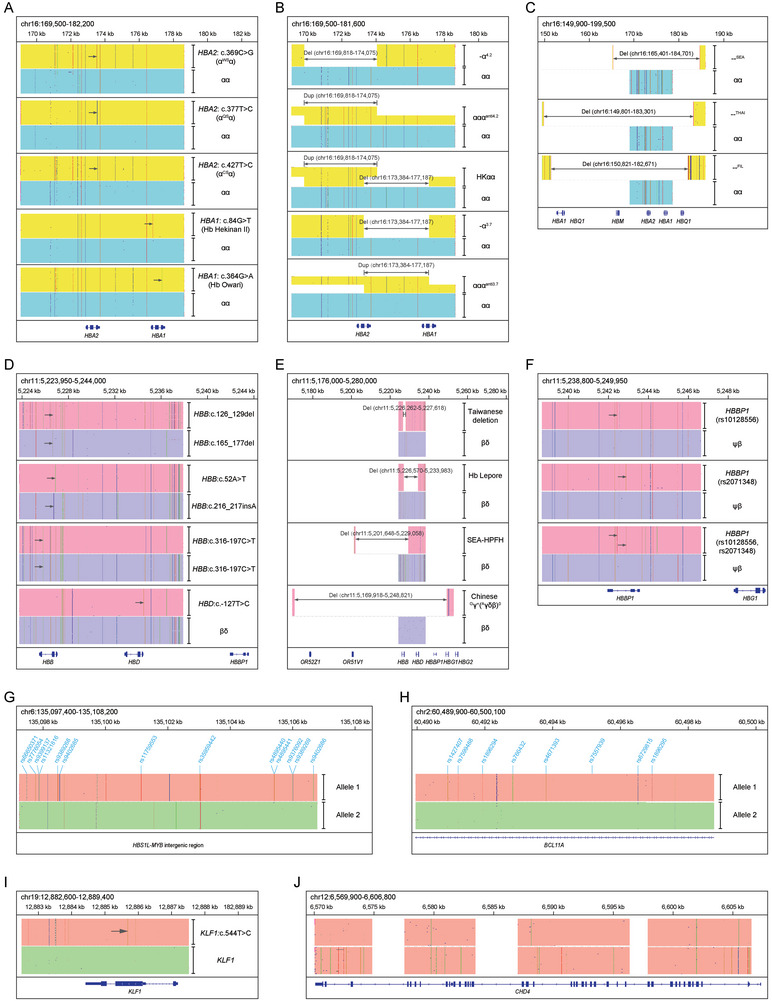
Integrative Genomics Viewer (IGV) plots displaying the long CCS reads of T‐LRS for representative samples. A) IGV plots displaying the detection of variants in *HBA2* (c.369C > G, c.377T > C, and c.427T > C) and *HBA1* (c.84G > T, and c.364G > A). B) IGV plots displaying the detection of deletions (−α^4.2^ and −α^3.7^), duplications (ααα^4.2^ and ααα^3.7^), and structural rearrangements (HKαα) caused by unequal crossover in the α‐globin locus. The exact deletion regions of −α^4.2^ and −α^3.7^ were annotated according to IthaID 301 and 300, respectively. C) IGV plots displaying the detection of large deletions including –^SEA^, –^THAI^, and –^FIL^ in the α‐globin locus. D) IGV plots displaying the detection of variants in *HBB* (c.52A > T, c.126_129del, c.165_177del, c.216_217insA, and c.316–197C > T) and *HBD* (c.−127T > C). E) IGV plots displaying the detection of large deletions including Taiwanese deletion, Hb Lepore, SEA‐HPFH, and Chinese Gγ + (Aγδβ)0 in the β‐globin locus. F) IGV plots displaying the detection the rs10128556 and rs2071348, as well as cis‐configuration of the two variants in *HBBP1*. G) IGV plot displaying the detection and cis‐configuration of reported modifying SNPs in *HBS1L*‐*MYB* intergenic region. H) IGV plot displaying the detection and cis‐configuration of reported modifying SNPs in intron 2 of *BCL11A*. I) IGV plot displaying the detection of the heterozygous variant *KLF1*: c.544T > C. J) IGV plot displaying the CCS reads of four *CHD4* amplicons. LRS could determine the phasing inside one amplicon, but could not determine the phasing among different amplicons.

To evaluate the accuracy of the T‐LRS panel for detecting thalassemia variants, validation of the T‐LRS panel for detecting thalassemia variants was performed on genomic DNA samples with known genotypes, including SNVs/indels in *HBA1* and *HBA2* (**Figure** **3**A), structural variations such as ααα^anti3.7^, ααα^anti4.2^, and HKαα (Figure 3B), large deletions in the α‐globin locus including ‐α^3.7^, ‐α^4.2^, –^SEA^, –^THAI^, and –^FIL^ (Figure 3C), SNVs/indels in *HBB* and *HBD* (Figure 3D), large deletions in the β‐globin locus including Taiwanese deletion, Hb Lepore, SEA‐HPFH (South‐East Asia type hereditary persistence of fetal hemoglobin), and Chinese Gγ(Aγδβ)0 (Figure 3E). T‐LRS is also able to phase common variants in the β‐globin gene cluster, taking rs10128556 and rs2071348 in *HBBP1* as an example (Figure 3F). Moreover, the long‐sequencing reads facilitate construction of the haplotypes of modifier genes (Figure 3G–J; Figure S5, Supporting Information) within one amplicon. Taken together, the established T‐LRS assay enabled comprehensive and accurate analysis of disease‐causing variants of thalassemia, variant identification and haplotype construction of modifier genes in the panel.

### 3.3 Distinguishing Highly Homologous Regions in the α‐ and γ‐Globin Genes

**Figure 4:**
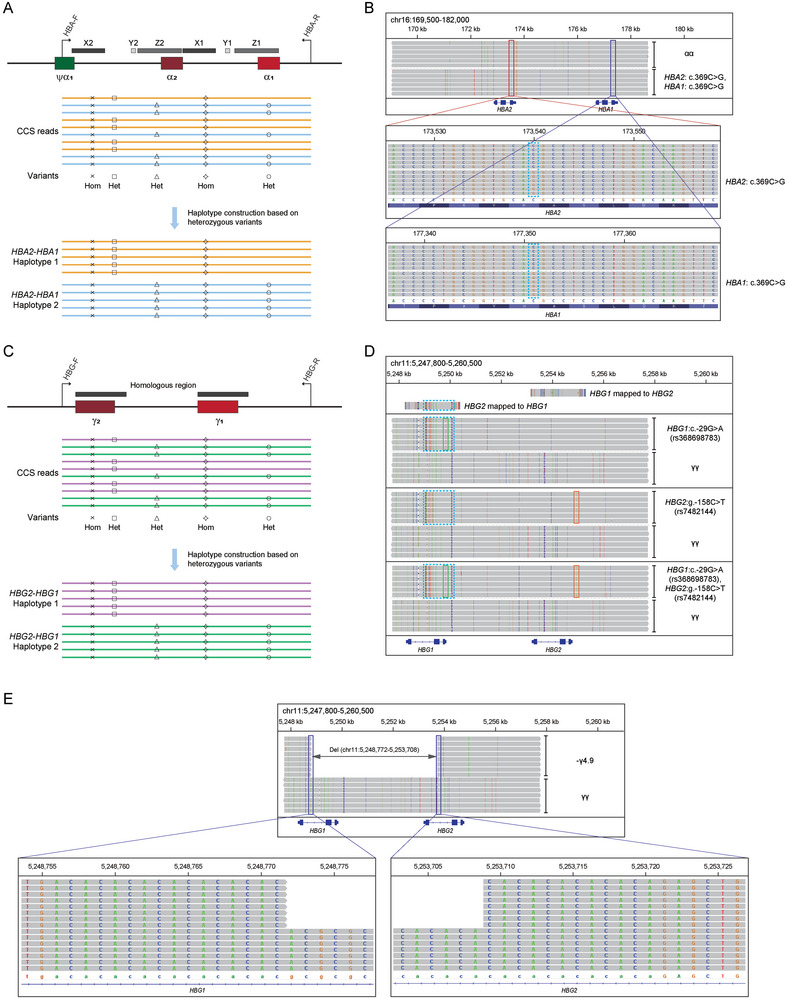
T‐LRS enabled precise variant calling and haplotype construction of the highly homologous regions in the α‐ and γ‐globin genes. A) Diagram showing the two 4 kb homologous units, design of primers, variant calling and haplotype construction based on heterozygous variants in the α‐globin genes. B) IGV plots displaying representative samples that had the variant c.369C > G in both *HBA1* and *HBA2* genes. C) Diagram showing the highly homologous regions, design of primers, variant calling and haplotype construction based on heterozygous variants in the γ‐globin genes. D) IGV plots displaying the detection of *HBG1*: c.−29G > A (blue box) and *HBG2*: g.−158C > T (orange box), as well as cis‐configuration of the two variants in γ‐globin gene loci. The blue box highlighted the conversion of *HBG1* to *HBG2* in the region encompassing promoter to intron 2. E) IGV plots displaying the 4.9 kb deletion between *HBG1* and *HBG2*, as well as the breakpoints identified by T‐LRS.

Genetic analysis of the two α‐globin genes is complex due to the presence of two highly homologous units spanning 4 kb, which are divided into X‐, Y‐, and Z‐homology boxes.^[^ ^41^ ^]^ Therefore, we designed the primers to capture the two homologous units in one long fragment, in order to accurately align and distinguish variants in *HBA1* and *HBA2* (Figure 3A). CCS reads were subjected to variant calling and heterozygous variants were used to divide the CCS reads into two clusters. The combination of variants in each cluster constructed the specific haplotype (Figure 3A). As a representative case, T‐LRS could accurately determine whether the variant c.369C > G was located in *HBA1*, *HBA2*, or in both genes (**Figures** **4**B, and S6, Supporting Information). Similarly, T‐LRS could distinguish the variants in highly homologous regions of γ‐globin genes allowing for haplotypic phasing (Figure 4C), such as the two known functional variants (c.−29G > A in *HBG1* and g.−158C > T in *HBG2*) associated with HbF (Figure 4D). As recombination between the homologous *HBG1* and *HBG2* could cause gene conversion and deletion, the T‐LRS panel identified a conversion in this region spanning from *HBG* promoter to intron 2 (Figure 4D), as well as a novel 4.9 kb deletion, with the precise breakpoint detected using the split reads (Figure 4E). In summary, T‐LRS enabled precise variant calling and haplotype construction of the highly homologous regions in the α‐ and γ‐globin genes.

### 3.4 Capability Performance of Thalassemic Variant Detection in a Cohort of 1020 β‐Thalassemia Patients

After optimization of the T‐LRS panel and test with samples with known genotypes, we next recruited a cohort of 1020 β‐thalassemia patients to test the capability performance of this panel in precise diagnosis of the disease. First, we performed routine PCR‐based approaches including reverse dot blotting and gap‐PCR, which covered a total of 23 mutations including SNVs, InDels, and SVs causing α‐ or β‐thalassemia (Table S1, Supporting Information). After genotyping by the routine PCR‐based approaches, the 1020 samples were then subjected to the T‐LRS panel, which facilitates the sequencing of both disease‐causing mutations of thalassemia and variants within the modifier genes mentioned above. As a result, T‐LRS panel was able to accurately detect all the thalassemia mutations called by routine PCR‐based approaches with 100% consistency (Table S2, Supporting Information). Those additional genotyping results by T‐LRS were further validated by multiplex ligation dependent probe amplification (MLPA) and Sanger sequencing (The representative results are shown in Figure S7, Supporting Information). In general, we additionally detected 17 HPFH alleles, 6 α‐duplication alleles, 24 rare α‐thalassemia mutation alleles, and 22 rare β‐thalassemia mutation alleles (File S2 and Table S3, Supporting Information). Upon detection of the patients carrying HPFH or α‐thalassemia mutations, we next evaluated the impact of these variants on the expression of HbF and age at first transfusion (Figure S8, Supporting Information). Notably, we found that the co‐inheritance of deletional α‐thalassemia mutations is more beneficial to ameliorating the clinical severity of β‐thalassemia patients compared to those of non‐deletional α‐thalassemia mutations as evaluated by ordinary one‐way analysis of variance (ANOVA) and Dunnett's correction for multiple comparisons (Figure S8, Supporting Information). Similarly, the patients with HPFH mutations showed relatively mild symptoms due to the high extent of re‐activation of HbF analyzed by unpaired Student's t‐test (Figure S8, Supporting Information). These results highlighted the need for comprehensive analysis of disease‐causing mutations of thalassemia.

### 3.5 Genetic Variants and Their Phenotypic Effects Identified in the Modifier Genes/Loci

In terms of the modifier genes or cis‐elements captured in this panel, we provided mutation spectra of the modifier genes in a large cohort of patients from Chinese population (Table S4, Supporting Information). We then carried out association studies to evaluate the effect of those variants on the expression of HbF, aiming at identifying novel modifier variants associated with the extent of re‐activation of HbF, which was one of key factors modulating the clinical heterogeneity of β‐thalassemia and sickle cell anemia. Consequently, we identified 198 variants statistically associated with HbF (false discovery rate, FDR < 0.05) (File S1, Supporting Information) while the FDR was determined by Benjamini‐Hochberg FDR correction for multiple testing using Plink 1.9.

### 3.6 Clinical Precision Diagnosis of β‐Thalassemia Patients by Integrated Analysis of Both Common and Rare Modifier Variants

**Figure 5:**
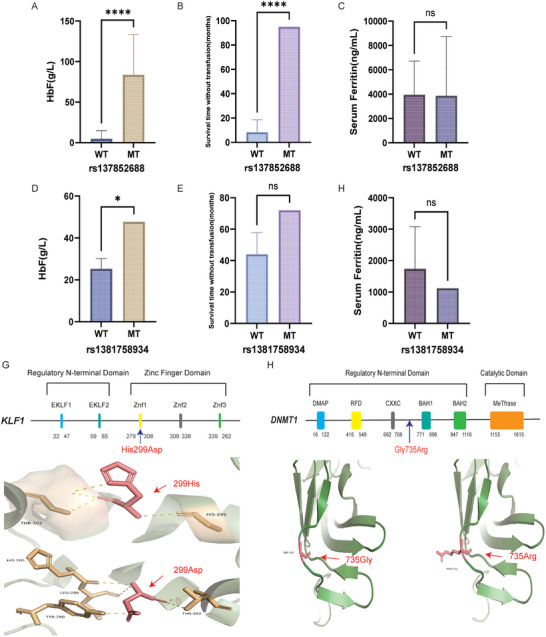
The effects of rare missense mutations in *KLF1* and *DNMT1* on the clinical severity of β‐thalassemia patients. A–C) The differences in the levels of HbF, survival time without transfusion and serum ferritin among the β‐thalassemia patients with different genotypes of rs137852688 in *KLF1*. D–F) The differences in the levels of HbF, survival time without transfusion and serum ferritin among the β‐thalassemia patients with different genotypes of rs1381758934 in *DNMT1*. G,H) The impact of the missense mutations in *KLF1* and *DNMT1* on protein structures modeled by Swiss‐prot and visualized in Pymol. The abbreviation of protein domains presented in Figure 5G,H were listed as below: DMAP: DMAP_binding; FRD: Cytosine specific DNA methyltransferase replication foci domain; CXXC: CXXC zinc finger domain; BAH1: Bromo adjacent homology (BAH) domain; BAH2: Bromo adjacent homology (BAH) domain; MeTfrase: C‐5 cytosine methyltransferase; EKLF1: Erythroid krueppel‐like transcription factor, transactivation 1; EKLF2: Erythroid krueppel‐like transcription factor, transactivation 2; Znf1: Zinc finger C2H2‐type domain; Znf2: Zinc finger C2H2‐type domain; Znf3: Zinc finger C2H2‐type domain. The levels of *p* values for the evaluation of statistical differences between the two groups were marked by asterisk. “*” means *p* < 0.05; “**” means *p* < 0.01; “***” means *p* < 0.001; “****” means *p* < 0.0001.

Among the 198 HbF‐associated variants, the majority of them were rare mutations with minor allele frequency (MAF) lower than 0.01 in this cohort. Notably, two rare missense variants were found with significant impact on the clinical severity of β‐thalassemia patients. The first one is NC_000019.10: g.12885335G > C (NP_006554.1:p.His299Asp) in *KLF1* marked as rs137852688 and the second one is NC_000019.10:g.10151412C > T (NP_001370.1:p.Gly735Arg) in *DNMT1* marked as rs1381758934. Of note, the first variant was known to be associated with HbF while the latter remains unknown of its correlation with HbF levels.^[^ ^20^ ^]^ The carriers of GC genotype in rs137852688 are two β^0^/β^0^ patients with abnormally high levels of HbF (48.50 and 118.87 g L^−1^, respectively). We therefore compared their key phenotypes with those patients with the same disease‐causing mutations of thalassemia. As a result, we found that the two patients exhibited higher levels of HbF, later age of onset reflected by the survival time without transfusion, but no significant changes in the levels of serum ferritin (**Figure** **5**A–C). Given the limited number of cases, a one‐sample t‐test was employed for the inter‐group comparison. Similar analysis was conducted on the rs1381758934 in *DNMT1* while we observed remarkable increase in the HbF levels (*p* = 0.038) and a trend of ameliorated clinical severity reflected by later age of onset and lower levels of serum ferritin with marginal *p* values (Figure 5D–F). We next explored the effects of these two functional mutations by homology modeling. Interestingly, we found that rs137852688 has led to an amino acid substitution of conserved residues in zinc finger domain 1 of *KLF1* (Figure 5G), which has been reported to be associated with In (Lu) phenotype,^[^ ^42^ ^]^ suggesting pleiotropy of this locus. Furthermore, the missense mutation in *DNMT1* was predicted to alter the state of its flanking α‐helix though it was not located with any of known domains (Figure 5H).

Upon identification of novel rare mutations associated with the expression of HbF, we next attempted to answer whether targeting and genotyping the modifier genes would better explain the clinical heterogeneity of β‐thalassemia patients. We thus evaluated the impact of three known modifier variants (rs4671393 in *BCL11A*, rs7776054 in *MYB‐HBS1L* intergenic regions, rs7482144 in *HBG* promoter and *KLF1* mutations) on the HbF levels and clinical typing of the 1020 β‐thalassemia patients. Notably, we found accumulated effects of the known variants on the clinical phenotypes of β‐thalassemia (Table S5, Supporting Information), which means that with the increasing number of “beneficial” variants one carries, the more likely this β‐thalassemia patient might show milder clinical symptoms with higher expression of HbF. These results suggest the practicability of the T‐LRS panel in precise diagnosis and phenotyping of β‐thalassemia.

### 3.7 Haplotype Clustering and Haplotype‐Based Association Analysis in *HBG1/HBG2* and *HBA1/HBA2* Homologous Regions

**Figure 6:**
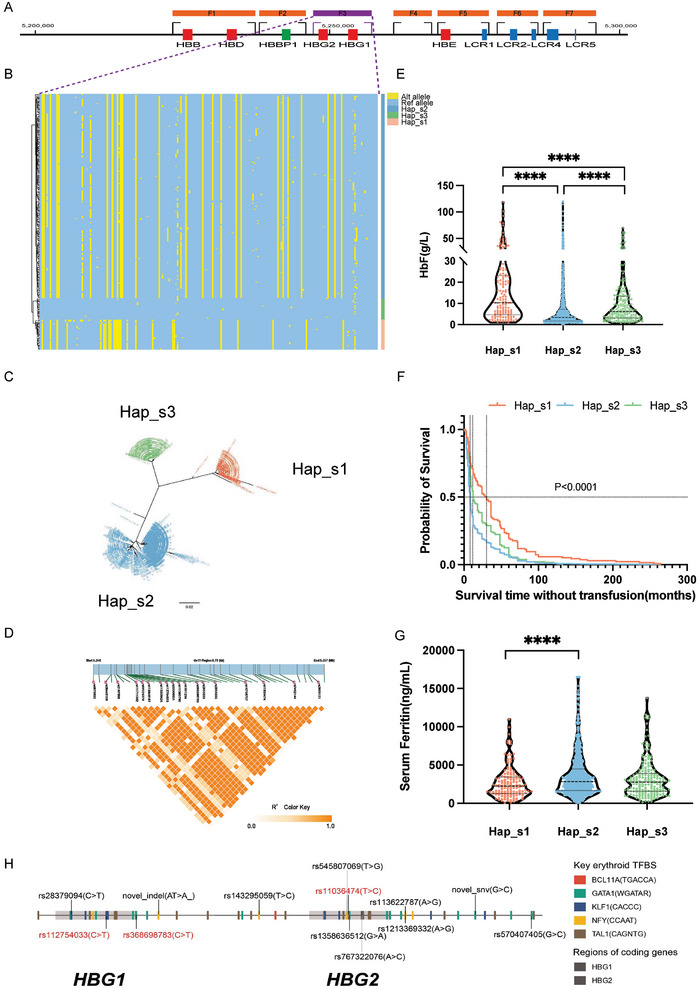
The general haplotypes of *HBG1/HBG2* regions and their phenotypic effects in the cohort of 1020 β‐thalassemia patients. (A) The diagram of seven fragments in the β‐globin gene cluster designed for long‐read sequencing in the T‐LRS panel. Among them, F3 covering the regions of *HBG1* and *HBG2* was highlighted by dotted purple lines to further show the unique haplotypes of the cohort in this fragment; (B) A heatmap displaying the haplotypes of the *HBG1/HBG2* haplotypes identified from 1020 β‐thalassemia patients. A total of 249 SNVs were identified from the 1020 β‐thalassemia patients in this region. Each row represents one haplotype while the 249 grids in each row, marked in either blue or red, denote the allele information in corresponding position. The blue color stands for a reference allele in this locus while the red stands for alteration allele. A total of 209 unique haplotypes were identified and these haplotypes were clustered into 3 main groups as shown in this figure; (C) The overview of the tree clustering results of the redundant 2040 *HBG1/HBG2* haplotypes in the patient population; (D) The LD block showing the linkage disequilibrium of the variants within the *HBG1/HBG2* genomic regions. The deeper color in each cell indicated higher R2 values, which means higher linkage extent between the two variants of interest; (E‐G) The effects of the three major haplotypes in the β‐thalassemia patients on the expression levels of HbF (E), the transfusion‐free survival time (F) and the levels of serum ferritin (G); H) A diagram showing the SNVs in the *HBG1‐HBG2* regions which potentially altered the transcription factor binding of 5 key erythroid regulators, namely *KLF1, BCL11A, GATA1, NFY, TAL1*. The consensus binding motifs of each TFs were shown on the right and their potential binding positions were highlighted in different colors on the horizontal bar standing for genomic region of *HBG1* and *HBG2*. 13 SNVs marked on this bar, which were identified as statistically significant with the expression of HbF, were predicted to alter the transcription binding of one of the 5 specific regulators mentioned above.

By clustering the CCS reads for each β‐thalassemia sample, the two alleles of *HBG1‐HBG2* region were distinguished and the variants called in each haplotype were marked. We thus yielded a matrix composed of 2040 haplotypes from the 1020 samples, which were then de‐duplicated according to the allele status and 249 unique haplotypes were finally obtained. These unique haplotypes were clustered into three groups, termed as Hap_s1, Hap_s2, and Hap_s3 (**Figure** **6**A–C), indicating the major haplotypes in *HBG1/HBG2* region existed in β‐thalassemia patients from southern China. The LD block was constructed to show the linkage of variants in this region (Figure 6D). Notably, we found that the patients carrying Hap_s1 showed significantly higher levels of HbF (mean value = 10.30 g L^−1^) than the patients with Hap_s2 and Hap_s3 (mean value of Hap_s2 = 3.39 g L^−1^, mean value of Hap_s2 = 6.10 g L^−1^) analyzed using unpaired t‐test (Figure 6E). We further confirmed that the elevated HbF levels associated with Hap_s1 are not due to a higher linkage with milder mutations of β‐thalassemia. This finding also suggests that Hap_s1 acts independently, driven by genetic variants within the HBG1/2 region that leads to increased levels of HbF (Figure S9, Supporting Information). The particularly high expression of HbF led to later age of onset for the patients in this group by survival analysis (Figure 6F) and lower levels of serum ferritin as well (Figure 6G). We next scanned all the variants identified from this cohort to identify 13 variants that potentially altered the binding motifs of key erythroid transcription factors (TFs) including *BCL11A, GATA1, KLF1, NFY*, and *TAL1* (Figure 6H), among which the three variants highlighted in red were from Hap_s1. These data might facilitate identification of functional or tag SNPs from the HbF‐associated haplotype.

Similar analysis on *HBA1/HBA2* homologous region were then conducted and we found 5 major haplotypes from the population of 1020 β‐thalassemia patients (Figure S10, Supporting Information). Interestingly, we identified one out of the 5 haplotypes significantly associated with higher levels of HbF and later age of onset (Figure S10, Supporting Information). This unique haplotype was closely linked with the three deletional types of α‐thalassemia including –^SEA^, ‐α^3.7^, and ‐α^4.2^, which ameliorated the α/β‐globin imbalance and resulted in the milder symptoms of the patients (Figure S11, Supporting Information). The haplotypic phasing and association studies might allow for a better understanding toward thalassemic patient‐specific haplotypes and its impacts on the clinical severity of β‐thalassemia.

## 4 Discussion

In this study, we first designed a T‐LRS panel capturing the α/β‐globin gene clusters and modifier genes or loci known to regulate hemoglobin switching or hematopoiesis. A cohort of 1020 β‐thalassemia patients was then recruited and subjected to the T‐LRS panel. Though this is a cohort enriched with β‐thalassemia patients, 158 of these patients were also in co‐inheritance with α‐thalassemia mutations, allowing simultaneous evaluation of detection accuracy of both α‐ and β‐thalassemia mutations for this T‐LRS panel. We performed the design of recruiting the patient‐based cohort aiming not only at evaluating the accuracy of this panel in detecting thalassemia mutations, but also trying to assess its practicability in precise phenotyping of β‐thalassemia and dissect the haplotypes of globin gene clusters enriched with disease‐causing alleles. The combined evaluation of both thalassemia mutations and modifier variants might promote individualized prenatal diagnosis and genetic counselling. Our previous efforts on at‐risk couple screening by LRS have demonstrated that a comprehensive detection of disease‐causing SNVs and CNVs of both α‐ and β‐thalassemia is fundamental in avoiding missed diagnosis of thalassemia major fetus.^[^ ^30^ ^]^

After verification of capability performance of the T‐LRS panel in detecting thalassemia mutations, we focused on the genetic variants within the modifier genes or loci in this cohort. In this study, we designed to capture 10 modifier genes or 3 major cis‐elements, which enabled observation of mutation spectra within these target regions among 1020 β‐thalassemia patients. By introducing the transfusion dependencies and HbF levels of the patients as dependent variables, we were able to launch phenotype‐genotype association studies and identify 198 variants significantly correlated with the phenotypes mentioned above. The findings could be divided into “known variants in known genes” as Tier 1 results and “novel variants in known genes” as Tier 2 results. Further analysis on Tier 1 results suggested that the patients tended to have higher levels of HbF and milder symptoms with the accumulation of the variants previously reported in *BCL11A, HMIP* and *HBG* gene promoters, making this T‐LRS panel a promising tool in both precise diagnosis and phenotyping of β‐hemoglobinopathies. In terms of Tier 2 results, we aimed to provide the mutation spectra on the genes known to participate in hemoglobin switching or hematopoiesis such as *CHD4, KLF3, KLF8* and *DNMT1*. The natural variants identified in a patient‐based population might serve as a good supplement in backing laboratorial findings by evaluating their impact on patients’ phenotypes despite the limitation that only a part of the modifier genes were included for economic reasons.^[^ ^43^ ^]^

Finally, we investigated the major haplotypes of the *HBG1/HBG2* and *HBA1/HBA2* in the β‐thalassemia cohort. The long sequencing reads spanning the homologous regions ensured unambiguous alignment of the reads as the putative length of amplification fragments reached 7122 bp for *HBG1/HBG2* and 8135 bp for *HBA1/HBA2*, which made the haplotype phasing more accurate compared to next‐generation sequencing (NGS), an approach with sequencing reads generally ranging from 80 to 150 bp, resulting in false mapping and reduced amounts of effective reads. According to the findings of genotype‐phenotype association analysis in this study, we propose that a combined panel for the detection of α/β‐globin gene clusters and key modifier genes including *BCL11A*, *MYB‐HBS1L*, *KLF1*, *HBG1/HBG2*, and *DNMT1* is capable for precise diagnosis of thalassemia while the average cost would be ≈50 US dollars per sample, which makes this method to have promising application prospects in the population screening of thalassemia. To our knowledge, this is the first population report on haplotypes of globin genes in a large‐scale β‐thalassemia patient cohort by T‐LRS, which also lead to the identification of the unique haplotypes associated with clinical phenotypes of β‐thalassemia. The population haplotype results might facilitate further studies on the identification of functional or tag SNPs among a series of highly linked variants.

## Conflict of Interest

The authors declare no conflict of interest.

## Author Contributions

Y.Y., C.N., and A.M. contributed equally to this work. Conceptualization was performed by Z.R. and X.X. Methodology was performed by Y.Y., C.N., A.M., J.Z., W.C., Q.Z., Y. Liu. Project administration was performed by B.L., K.L., and X.H. Investigation was performed by Q.Z., Y.Y., L.Q., L.L., J. Cai, R.L., X. Zhang, L.Z., Y. Liu, B.H., H.Q., Y. Huang, Z.H., J.L., X.Q., J. Chen, X.F., Q.L., W.L., X. Zhou, Y. Liang, X. Long, J.Q., L. Yan, W.Z., L. Yu, C.F., D.T., T.Z., J.T. Funding acquisition: XX. Original draft was written by Y.Y., C.N., and A.M. Review was written and editing was performed by Z.R. and X.X.

## Supporting information



Supporting Information

Supplemental File 1

Supplemental File 2

Supplemental File 3

## Data Availability

The data that support the findings of this study are available on request from the corresponding author. The data are not publicly available due to privacy or ethical restrictions.

## References

[1] H. L. Muncie Jr. , J. Campbell , Am. Fam. Physician 2009, 80, 339. 19678601

[2] A. Kattamis , J. L. Kwiatkowski , Y. Aydinok , Lancet 2022, 399, 2310. 35691301 10.1016/S0140-6736(22)00536-0

[3] M. Cazzalo , Blood 2022, 139, 2460.34932791

[4] A. T. Taher , A. N. Saliba , Hematology Am. Soc. Hematol. Educ. Program 2017, 2017, 265. 29222265 10.1182/asheducation-2017.1.265 PMC6142532

[5] M. U. Muckenthaler , S. Rivella , M. W. Hentze , B. Galy , Cell 2017, 168, 344. 28129536 10.1016/j.cell.2016.12.034 PMC5706455

[6] I. Motta , R. Bou‐Fakhredin , A. T. Taher , M. D. Cappellini , Drugs 2020, 80, 1053. 32557398 10.1007/s40265-020-01341-9 PMC7299245

[7] J. Traeger‐Synodinos , C. L. Harteveld , J. M. Old , M. Petrou , R. Galanello , P. Giordano , M. Angastioniotis , B. De la Salle , S. Henderson , A. May , Eur. J. Hum. Genet. 2015, 23, 426. 25052315 10.1038/ejhg.2014.131 PMC4666573

[8] V. Viprakasit , S. Ekwattanakit , Hematol. Oncol. Clin. North Am. 2018, 32, 193. 29458726 10.1016/j.hoc.2017.11.006

[9] F. B. Piel , D. J. Weatherall , N. Engl. J. Med. 2014, 371, 1908. 25390741 10.1056/NEJMra1404415

[10] X. Shang , Z. Peng , Y. Ye , Asan , X. Zhang , Y. Chen , B. Zhu , W. Cai , S. Chen , R. Cai , X. Guo , C. Zhang , Y. Zhou , S. Huang , Y. Liu , B. Chen , S. Yan , Y. Chen , H. Ding , X. Yin , L. Wu , J. He , D. Huang , S. He , T. Yan , X. Fan , Y. Zhou , X. Wei , S. Zhao , D. Cai , F. Guo , et al., E. Bio. Medicine 2017, 23, 150.

[11] D. Songdej , P. Kadegasem , N. Tangbubpha , W. Sasanakul , B. Deelertthaweesap , A. Chuansumrit , N. Sirachainan , Br. J. Haematol. 2022, 198, 1051. 35819869 10.1111/bjh.18356

[12] W. Huang , S. Qu , Q. Qin , X. Yang , W. Han , Y. Lai , J. Chen , S. Zhou , X. Yang , W. Zhou , Clin. Chem. 2023, 69, 1062. 37311260 10.1093/clinchem/hvad073

[13] S. Lunke , S. E. Bouffler , C. V. Patel , S. A. Sandaradura , M. Wilson , J. Pinner , M. F. Hunter , C. P. Barnett , M. Wallis , B. Kamien , T. Y. Tan , M. L. Freckmann , B. Chong , D. Phelan , D. Francis , K. S. Kassahn , T. Ha , S. Gao , P. Arts , M. R. Jackson , H. S. Scott , S. Eggers , S. Rowley , K. Boggs , A. Rakonjac , G. R. Brett , M. G. de Silva , A. Springer , Nat. Med. 2023, 29, 1681. 37291213 10.1038/s41591-023-02401-9 PMC10353936

[14] C. Chen , R. Li , J. Sun , Y. Zhu , L. Jiang , J. Li , F. Fu , J. Wan , F. Guo , X. An , Y. Wang , L. Fan , Y. Sun , X. Guo , S. Zhao , W. Wang , F. Zeng , Y. Yang , P. Ni , Y. Ding , B. Xiang , Z. Peng , C. Liao , Genome Med. 2021, 13, 18. 33546747 10.1186/s13073-021-00836-8 PMC7866698

[15] R. Karnpean , W. Tepakhan , P. Suankul , S. Thingphom , A. Poonsawat , N. Thanunchaikunlanun , R. Ruangsanngamsiri , W. Jomoui , Genes (Basel) 2022, 13, 1384. 36011295 10.3390/genes13081384 PMC9407504

[16] D. Porubsky , E. E. Eichler , Cell 2024, 187, 1024. 38290514 10.1016/j.cell.2024.01.002 PMC10932897

[17] P. Hariharan , A. Nadkarni , Blood Rev. 2021, 49, 100823. 33726930 10.1016/j.blre.2021.100823

[18] H. T. Bae , C. T. Baldwin , P. Sebastiani , M. J. Telen , A. Ashley‐Koch , M. Garrett , W. C. Hooper , C. J. Bean , M. R. DeBaun , D. E. Arking , P. Bhatnagar , J. F. Casella , J. R. Keefer , E. Barron‐Casella , V. Gordeuk , G. J. Kato , C. Minniti , J. Taylor , A. Campbell , L. Luchtman‐Jones , C. Hoppe , M. T. Gladwin , Y. Zhang , M. H. Steinberg , Blood 2012, 120, 1961. 22936743 10.1182/blood-2012-06-432849 PMC3433099

[19] J. Makani , S. Menzel , S. Nkya , S. E. Cox , E. Drasar , D. Soka , A. N. Komba , J. Mgaya , H. Rooks , N. Vasavda , G. Fegan , C. R. Newton , M. Farrall , S. L. Thein , Blood 2011, 117, 1390. 21068433 10.1182/blood-2010-08-302703 PMC5555384

[20] D. Liu , X. Zhang , L. Yu , R. Cai , X. Ma , C. Zheng , Y. Zhou , Q. Liu , X. Wei , L. Lin , T. Yan , J. Huang , N. Mohandas , X. An , X. Xu , Blood 2014, 124, 803. 24829204 10.1182/blood-2014-03-561779 PMC4118488

[21] D. Chen , Y. Zuo , X. Zhang , Y. Ye , X. Bao , H. Huang , W. Tepakhan , L. Wang , J. Ju , G. Chen , M. Zheng , D. Liu , S. Huang , L. Zong , C. Li , Y. Chen , C. Zheng , L. Shi , Q. Zhao , Q. Wu , S. Fucharoen , C. Zhao , X. Xu , Am. J. Hum. Genet. 2017, 101, 130. 28669403 10.1016/j.ajhg.2017.05.012 PMC5501772

[22] M. Amaya , M. Desai , M. N. Gnanapragasam , S. Z. Wang , S. Zu Zhu , D. C. Williams Jr. , G. D. Ginder , Blood 2013, 121, 3493. 23444401 10.1182/blood-2012-11-466227 PMC3637018

[23] D. S. Vinjamur , Q. Yao , M. A. Cole , C. McGuckin , C. Ren , J. Zeng , M. Hossain , K. Luk , S. A. Wolfe , L. Pinello , D. E. Bauer , Nat. Genet. 2021, 53, 719. 33859416 10.1038/s41588-021-00843-w PMC8180380

[24] R. Feng , T. Mayuranathan , P. Huang , P. A. Doerfler , Y. Li , Y. Yao , J. Zhang , L. E. Palmer , K. Mayberry , G. E. Christakopoulos , P. Xu , C. Li , Y. Cheng , G. A. Blobel , M. C. Simon , M. J. Weiss , Nature 2022, 610, 783. 36224385 10.1038/s41586-022-05312-w PMC9773321

[25] Y. Wu , J. Zeng , B. P. Roscoe , P. Liu , Q. Yao , C. R. Lazzarotto , K. Clement , M. A. Cole , K. Luk , C. Baricordi , A. H. Shen , C. Ren , E. B. Esrick , J. P. Manis , D. M. Dorfman , D. A. Williams , A. Biffi , C. Brugnara , L. Biasco , C. Brendel , L. Pinello , S. Q. Tsai , S. A. Wolfe , D. E. Bauer , Nat. Med. 2019, 25, 776. 30911135 10.1038/s41591-019-0401-y PMC6512986

[26] P. Antoniou , G. Hardouin , P. Martinucci , G. Frati , T. Felix , A. Chalumeau , L. Fontana , J. Martin , C. Masson , M. Brusson , G. Maule , M. Rosello , C. Giovannangeli , V. Abramowski , J. P. de Villartay , J. P. Concordet , F. Del Bene , W. El Nemer , M. Amendola , M. Cavazzana , A. Cereseto , O. Romano , A. Miccio , Nat. Commun. 2022, 13, 6618. 36333351 10.1038/s41467-022-34493-1 PMC9636226

[27] H. Frangoul , D. Altshuler , M. D. Cappellini , Y. S. Chen , J. Domm , B. K. Eustace , J. Foell , J. de la Fuente , S. Grupp , R. Handgretinger , T. W. Ho , A. Kattamis , A. Kernytsky , J. Lekstrom‐Himes , A. M. Li , F. Locatelli , M. Y. Mapara , M. de Montalembert , D. Rondelli , A. Sharma , S. Sheth , S. Soni , M. H. Steinberg , D. Wall , A. Yen , S. Corbacioglu , N. Engl. J. Med. 2021, 384, 252. 33283989 10.1056/NEJMoa2031054

[28] T. Mayuranathan , G. A. Newby , R. Feng , Y. Yao , K. D. Mayberry , C. R. Lazzarotto , Y. Li , R. M. Levine , N. Nimmagadda , E. Dempsey , G. Kang , S. N. Porter , P. A. Doerfler , J. Zhang , Y. Jang , J. Chen , H. W. Bell , M. Crossley , S. V. Bhoopalan , A. Sharma , J. F. Tisdale , S. M. Pruett‐Miller , Y. Cheng , S. Q. Tsai , D. R. Liu , M. J. Weiss , J. S. Yen , Nat. Genet. 2023, 55, 1210. 37400614 10.1038/s41588-023-01434-7 PMC10722557

[29] L. Xu , A. Mao , H. Liu , B. Gui , K. W. Choy , H. Huang , Q. Yu , X. Zhang , M. Chen , N. Lin , L. Chen , J. Han , Y. Wang , M. Zhang , X. Li , D. He , Y. Lin , J. Zhang , D. S. Cram , H. Cao , J. Mol. Diagn. 2020, 22, 1087. 32473995 10.1016/j.jmoldx.2020.05.004

[30] Q. Liang , J. He , Q. Li , Y. Zhou , Y. Liu , Y. Li , L. Tang , S. Huang , R. Li , F. Zeng , A. Mao , Y. Liu , D. Liang , L. Wu , Clin. Chem. 2023, 69, 239. 36683393 10.1093/clinchem/hvac200

[31] Q. Liang , W. Gu , P. Chen , Y. Li , Y. Liu , M. Tian , Q. Zhou , H. Qi , Y. Zhang , J. He , Q. Li , L. Tang , J. Tang , Y. Teng , Y. Zhou , S. Huang , Z. Lu , M. Xu , W. Hou , T. Huang , Y. Li , R. Li , L. Hu , S. Li , Q. Guo , Z. Zhuo , Y. Mou , D. S. Cram , L. Wu , J. Mol. Diagn. 2021, 23, 1195. 34293487 10.1016/j.jmoldx.2021.06.008

[32] S. Li , X. Han , Y. Xu , C. Chang , L. Gao , J. Li , Y. Lu , A. Mao , Y. Wang , J. Mol. Diagn. 2022, 24, 1009. 35659528 10.1016/j.jmoldx.2022.05.001

[33] Y. Liu , M. Chen , J. Liu , A. Mao , Y. Teng , H. Yan , H. Zhu , Z. Li , D. Liang , L. Wu , Clin. Chem. 2022, 68, 927. 35714169 10.1093/clinchem/hvac046

[34] Y. Liu , D. Li , D. Yu , Q. Liang , G. Chen , F. Li , L. Gao , Z. Li , T. Xie , L. Wu , A. Mao , L. Wu , D. Liang , Thromb. Haemost. 2023, 123, 1151. 37285902 10.1055/a-2107-0702 PMC10686748

[35] J. Ye , S. McGinnis , T. L. Madden , Nucleic Acids Res. 2006, 34, W6. 16845079 10.1093/nar/gkl164 PMC1538791

[36] B. Giardine , J. Borg , E. Viennas , C. Pavlidis , K. Moradkhani , P. Joly , M. Bartsakoulia , C. Riemer , W. Miller , G. Tzimas , H. Wajcman , R. C. Hardison , G. P. Patrinos , Nucleic Acids Res. 2014, 42, D1063. 24137000 10.1093/nar/gkt911 PMC3964999

[37] P. Kountouris , C. W. Lederer , P. Fanis , X. Feleki , J. Old , M. Kleanthous , PLoS One 2014, 9, e103020. 25058394 10.1371/journal.pone.0103020 PMC4109966

[38] S. Richards , N. Aziz , S. Bale , D. Bick , S. Das , J. Gastier‐Foster , W. W. Grody , M. Hegde , E. Lyon , E. Spector , K. Voelkerding , H. L. Rehm , A. L. Q. A , Genet. Med. 2015, 17, 405. 25741868 10.1038/gim.2015.30 PMC4544753

[39] M. Martin , P. Ebert , T. Marschall , Methods Mol. Biol. 2023, 2590, 127. 36335496 10.1007/978-1-0716-2819-5_8

[40] T. Schwede , J. Kopp , N. Guex , M. C. Peitsch , Nucleic Acids Res. 2003, 31, 3381. 12824332 10.1093/nar/gkg520 PMC168927

[41] S. Farashi , C. L. Harteveld , Blood Cells Mol. Dis. 2018, 70, 43. 29032940 10.1016/j.bcmd.2017.09.004

[42] B. K. Singleton , N. M. Burton , C. Green , R. L. Brady , D. J. Anstee , Blood 2008, 112, 2081. 18487511 10.1182/blood-2008-03-145672

[43] K. J. Karczewski , L. C. Francioli , G. Tiao , B. B. Cummings , J. Alfoldi , Q. Wang , R. L. Collins , K. M. Laricchia , A. Ganna , D. P. Birnbaum , L. D. Gauthier , H. Brand , M. Solomonson , N. A. Watts , D. Rhodes , M. Singer‐Berk , E. M. England , E. G. Seaby , J. A. Kosmicki , R. K. Walters , K. Tashman , Y. Farjoun , E. Banks , T. Poterba , A. Wang , C. Seed , N. Whiffin , J. X. Chong , K. E. Samocha , E. Pierce‐Hoffman , et al., Nature 2020, 581, 434. 32461654 10.1038/s41586-020-2308-7 PMC7334197

