# Supplementary material for: Haplotype‐Resolved Genotyping and Association Analysis of 1,020 β‐Thalassemia Patients by Targeted Long‐Read Sequencing
Source: Adv Sci (Weinh). 2024 Dec 31;12(9):2410992. doi: 10.1002/advs.202410992 (PMC11884621; doi:10.1002/advs.202410992)

**Haplotype-resolved genotyping and association analysis of 1,020 β-thalassemia patients by targeted long-read sequencing**

**Supplemental materials**

**Figure S1.** Design of the PCR primers for the T-LRS panel. (A) Primer pair included in reaction 2 for the *GATA1* amplicon to cover exon 2 to exon 6. (B) Primer pair included in reaction 2 for the *GATAD2A* amplicon to cover exon 2 for the variant c.19C>T. (C) Primer pair included in reaction 2 for the *ZBTB7A* amplicon to cover the coding exons. (D) Primer pair included in reaction 2 for the *DNMT1* amplicon to cover exon 24 to exon 28 for the variant c.2633G>A. (E) Two primer pairs included in reaction 4 for the two *KLF3* amplicons to cover coding exons and all the introns. (F) Three primer pairs included in reaction 4 for the three *KLF8* amplicons to cover exon 2 to exon 8. (G) Two primer pairs included in reaction 4 for the two *SIRT1* amplicons to cover exon 1 to exon 9.


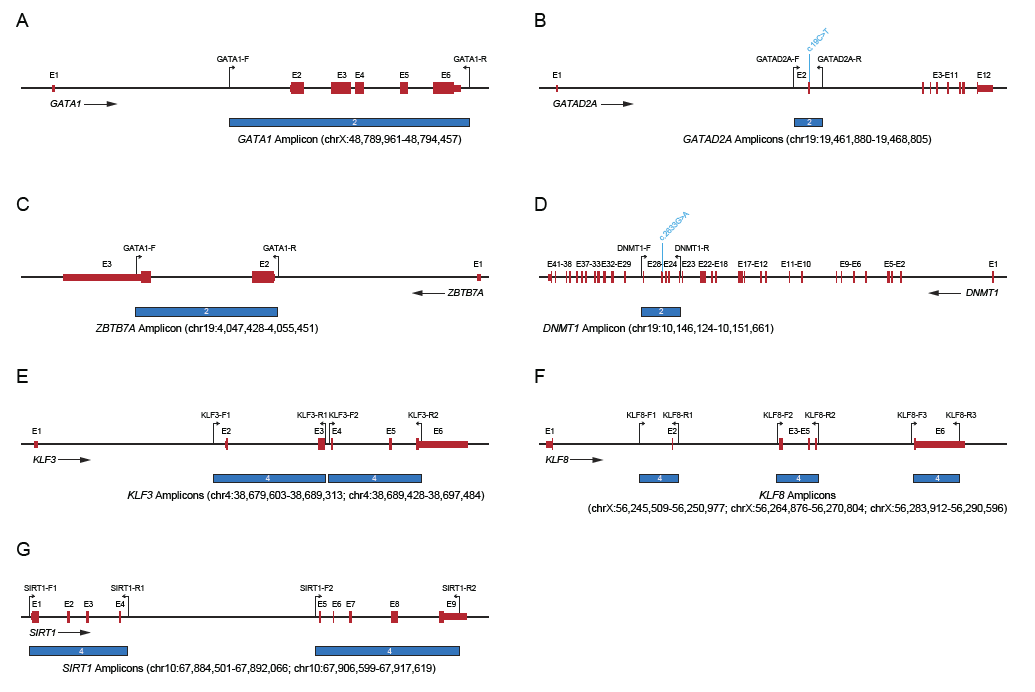


**Figure S2.** Confirmation of the multiplex LR-PCR reactions in T-LRS assay. (A) Examples of the amplicon products of different thalassemia genotypes in reaction 1. (B) Representative gel electrophoresis image of the 10, 10 and 9 amplicons in reaction 2, 3 and 4, respectively. Reaction 2 contained 10 fragments from up to bottom: *HBZP1* (10298 bp), *HS40* (9302 bp), *BCL11A* (8840bp), *ZBTB7A* (8024 bp), *GATAD2A* (6926 bp), *HBQ1* (5936 bp), *DNMT1* (5538 bp), *HBZ* (5206 bp), *GATA1* (4497 bp), and *HBM* (3960 bp). Reaction 3 contained 8 fragments from up to bottom: *HBG1-2* (9967 bp), *HBS1L-MYB* (9559 bp), *LCR4-5* (8996 bp), *HBE1-LCR1* (8442 bp), *HBBP1* (7957 bp), *LCR2-3* (6970 bp), *HBG2-HBE1* (6503 bp), and *KLF1* (5038 bp). Reaction 3 contained 11 fragments from up to bottom: *SIRT1-2* (11021 bp), *KLF3-1* (9711 bp), *CHD4-3* (9299 bp), *CHD4-4* (8813 bp), *KLF3-2* (8057 bp), *SIRT1-1* (7566 bp), *KLF8-3* (6685 bp), *CHD4-2* (6121 bp), *KLF8-2* (5929 bp), *KLF8-1* (5469 bp), and *CHD4-1* (4802 bp). *, specific amplicon; x, non-specific amplicon.


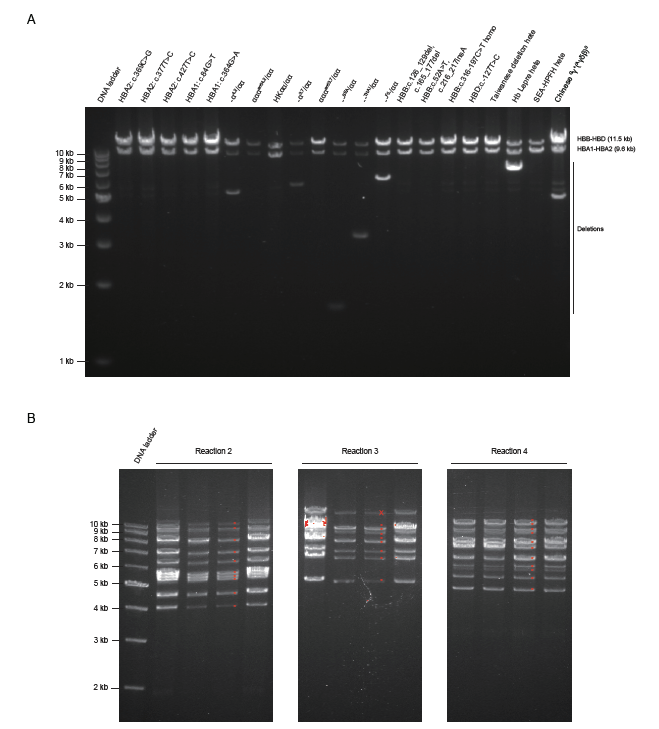


**Table S1.** List of common thalassemia mutations which are routinely detected in clinical practice of hospitals in Southern China

| Type of thalassemia | Common names | HGVS names |
| --- | --- | --- |
| Point mutations of  β-thalassemia | CD41-42 (-TCTT) |  |
|  | IVS-II-654 (C>T) |  |
|  | –28 (A > G) |  |
|  | CD 71/72 (+A) |  |
|  | CD 17 (AAG > TAG) |  |
|  | CD 26 (GAG>AAG) |  |
|  | CD43 (GAG>TAG) |  |
|  | –29 (A > G) |  |
|  | CD31 (−C) |  |
|  | –32 (C > A) |  |
|  | IVS-I-1 (G > T) |  |
|  | CD 27/28 (+C) |  |
|  | –30 (T > C) |  |
|  | CD 14/15 (+G) |  |
|  | Cap+ 40–43 (−AAAC) |  |
|  | initiation codon (ATG > AGG) |  |
|  | IVS-I-5 (G > C) |  |
| Deletional mutations of α-thalassemia | --^SEA^/αα |  |
|  | -α^3.7^/αα |  |
|  | -α^4.2^/αα |  |
| Non-deletional mutations of α-thalassemia | α^CS^α/αα |  |
|  | α^QS^α/αα |  |
|  | α^WS^α/αα |  |

**Figure S3.** Flowchart showing the design and bioinformatic pipeline of the T-LRS approach.


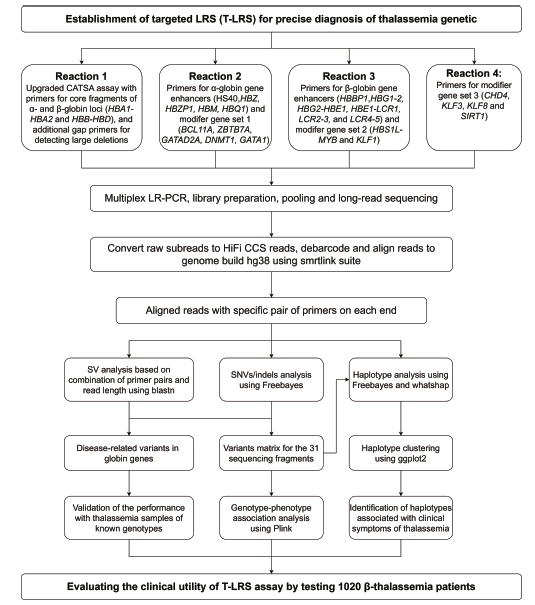


**Figure S4.** Flowchart showing the optimization of the T-LRS bioinformatic pipeline through SNVs/indels calling, variant database construction, and linked-variant haplotype analysis.


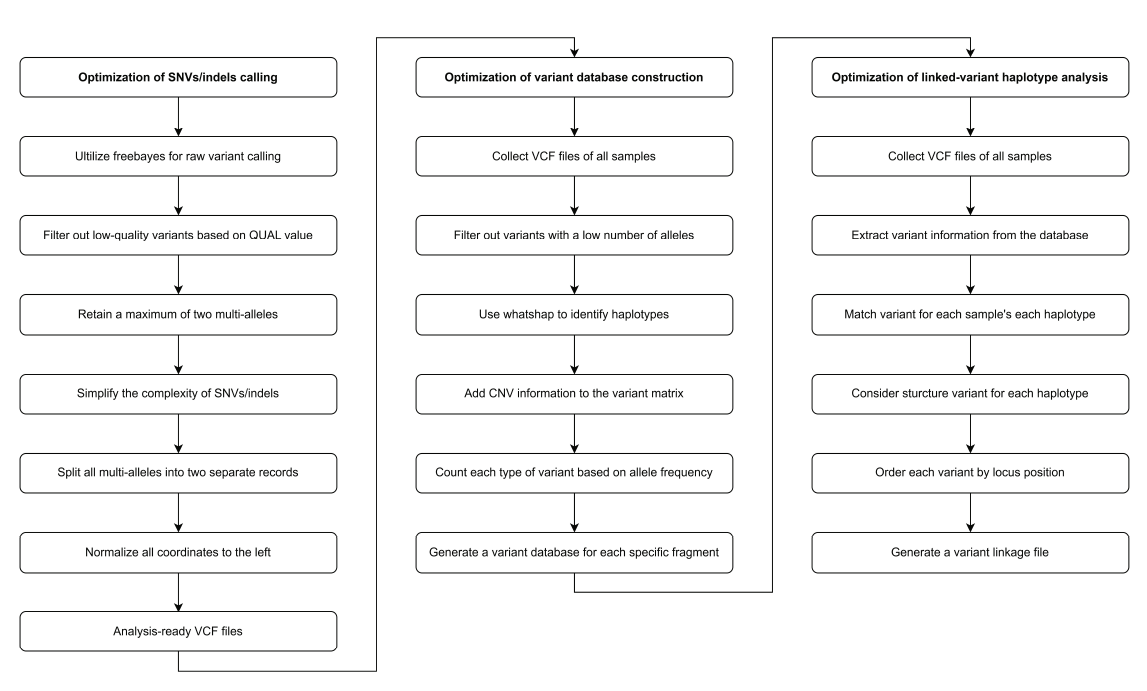


**Figure S5.** Representative IGV plots displaying the CCS reads of amplicons for *GATA1* (A), *GATAD2A* (B), *ZBTB7A* (C), *DNMT1* (D), *KLF3* (E), *KLF8* (F), and *SIRT1* (G).


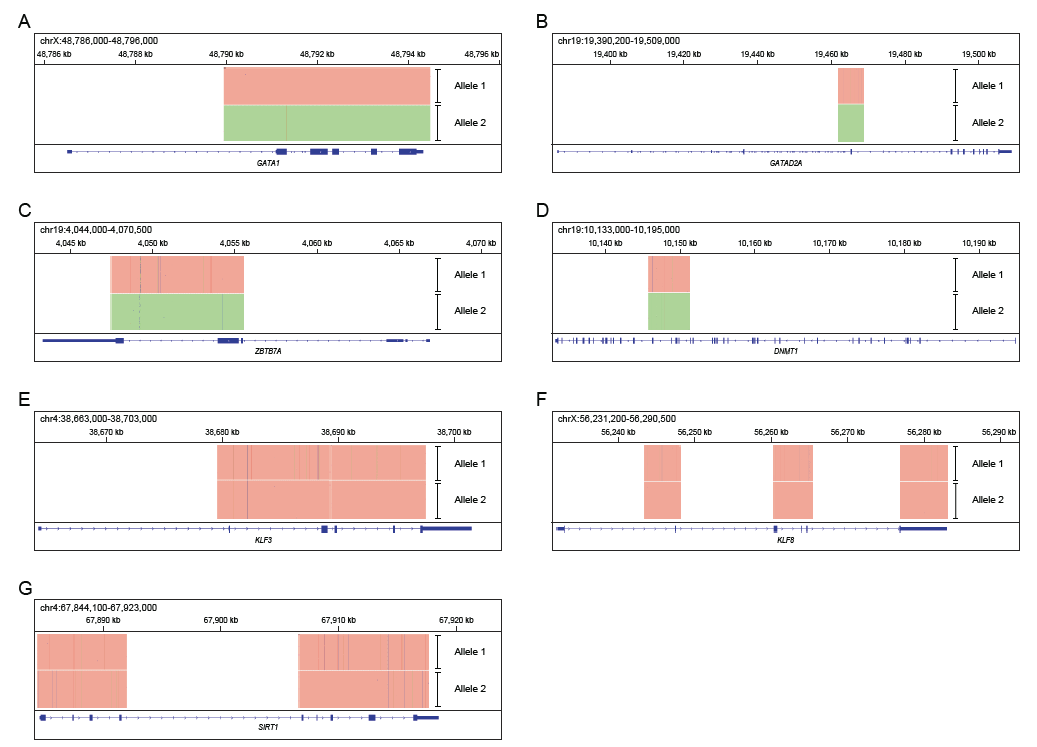


**Figure S6.** Representative IGV plots showing that LRS enabled discrimination of variant in *HBA2* and *HBA1*.


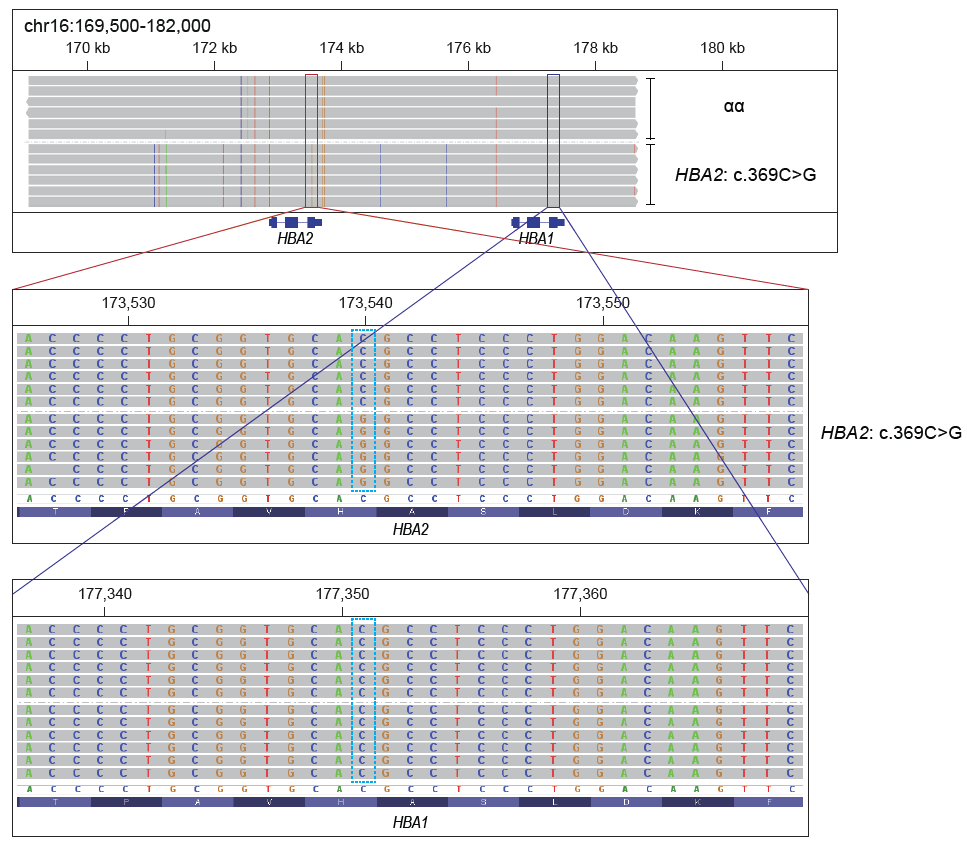


**Table S2.** The types and allele number of rare thalassemia mutations additionally detected by the T-LRS panel.

| Mutation type | Common mutation detected by both T-LRS  and routine diagnostic approaches  (N=allele number) | | | Additional rare mutations detected by T-LRS  and validated PCR approaches  (N=allele number) | | |  |
| --- | --- | --- | --- | --- | --- | --- | --- |
|  | T-LRS | Routine | Consistency | T-LRS | PCR re-typed | Consistency |  |
| β-thal point mutations | 1992 | 1992 | 100% | 23 | 23 | 100% | |
| α-thal point mutations | 27 | 27 | 100% | 20 | 20 | 100% | |
| α-thal deletions  or α-duplications | 137 | 137 | 100% | 11 | 11 | 100% | |
| Deletional HPFH | - | - | - | 14 | 14 | 100% | |

**Figure S7.** Representative diagrams of complex structural variants identified in both α- and β-globin gene clusters among the 1020 β-thalassemia patients. Panel A represents a β^0^/β^0^ thalassemia patient heterozygous in a --^Thai^ deletion in α-globin gene clusters, which was validated by MLPA (Panel C) and showed consistent breakpoint in both approaches; Panel B represents β^0^/β^0^ thalassemia patient heterozygous in a duplication in α-globin gene clusters (ααα^3.7^/αα), validated by MLPA (Panel D).


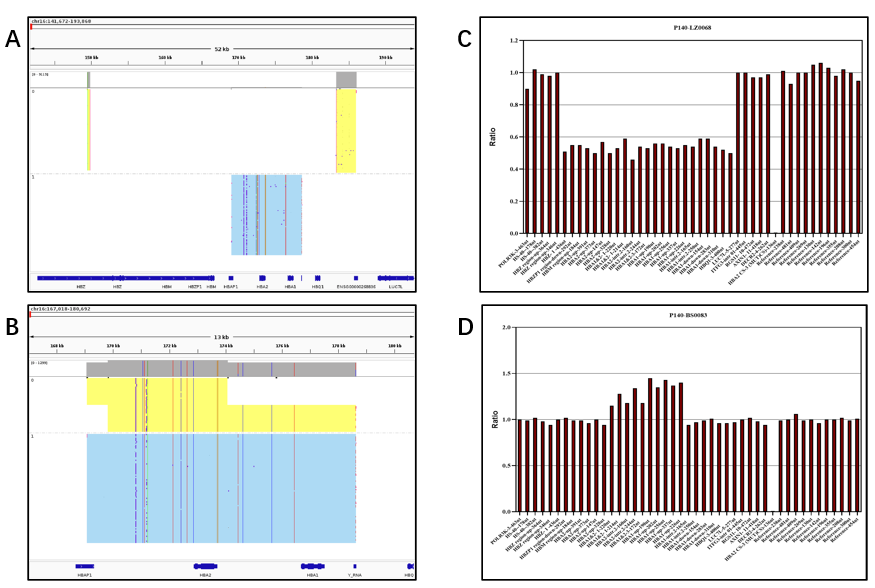


**Table S3.** The types and allele count of rare thalassemia mutations additionally detected by the T-LRS panel.

| Thalassemia type | Mutation names | | Allele counts by T-LRS | | Consistency with re-typed approaches |
| --- | --- | --- | --- | --- | --- |
| α-thal point mutation | | CD27 | | 5 | 100% |
|  |  | CD30(-GAG) | | 2 | 100% |
|  |  | CD121 | | 1 | 100% |
|  |  | IVS-I-11 (-24bp) | | 2 | 100% |
|  |  | IVS-II-34 (G>A) | | 1 | 100% |
|  |  | IVS-II-55 | | 3 | 100% |
|  |  | IVS-II-118 | | 1 | 100% |
|  |  | IVS-II-119 | | 1 | 100% |
|  |  | HBA2:c.2delT | | 1 | 100% |
|  |  | HBA2:c.40G>T | | 1 | 100% |
|  |  | HBA2:c.46G>A | | 1 | 100% |
|  |  | HBA2:c.300+34G>A | | 2 | 100% |
| β-thal point mutation | | -90（C>T) | | 2 | 100% |
|  |  | CD30(AGG>GGG) | | 2 | 100% |
|  |  | CD37(TGG>TAG) | | 3 | 100% |
|  |  | CD54-58(-TATGGGCAACCCT) | | 2 | 100% |
|  |  | CD95(+A) | | 1 | 100% |
|  |  | CD104 | | 1 | 100% |
|  |  | IVS-I-128(T>G) | | 1 | 100% |
|  |  | IVS-II-1 | | 1 | 100% |
|  |  | IVS-II-5(G>C) | | 6 | 100% |
|  |  | β nt 1587（A>G) | | 1 | 100% |
|  |  | Hb-Lepore | | 2 | 100% |
| α-thal SV | | -α^2.4^ | | 1 | 100% |
|  |  | ααα3.7 | | 4 | 100% |
|  |  | ααα4.2 | | 2 | 100% |
|  |  | HKαα | | 2 | 100% |
|  |  | --^THAI^ | | 2 | 100% |
| Deletional HPFH | | Chinese | | 10 | 100% |
|  |  | SEA-HPFH | | 4 | 100% |
|  | | Taiwanese | | 1 | 100% |

**Figure S8.** The impact of deletional/non-deletional α-thalassemia mutations and HPFH mutations on the clinical phenotypes of β-thalassemia patients. (A-B) The impact of deletional/non-deletional α-thalassemia mutations on the expression levels of HbF (A) and age at first transfusion (B) of β-thalassemia patients; (C-D) The impact of HPFH mutations on the expression levels of HbF (C) and age at first transfusion (D) of β-thalassemia patients;


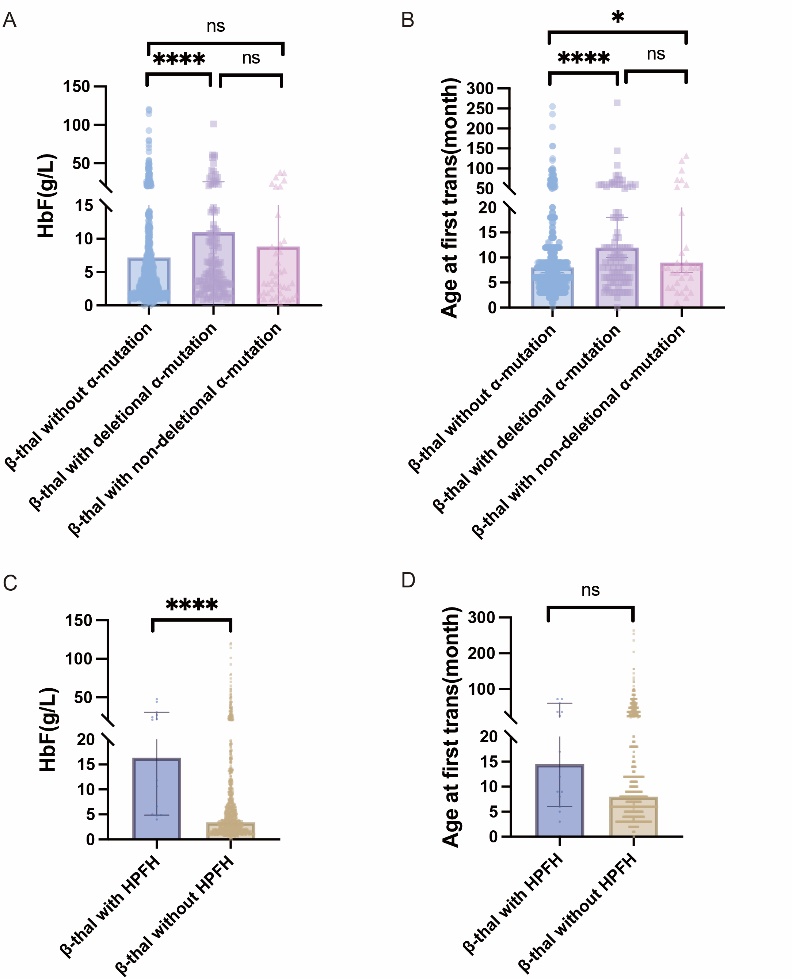


**Table S4.** The number and functional annotation of the genetic variants within the modifier variants

| Mutation types/Gene names | BCL11A | CHD4 | DNMT1 | GATA1 | GATAD2A | HBS1L | KLF3 | KLF8 | NOP2 | SIRT1 | ZBTB7A |
| --- | --- | --- | --- | --- | --- | --- | --- | --- | --- | --- | --- |
| Intron_Variant | 110 | 262 | 77 | 25 | 84 | 68 | 213 | 0 | 0 | 218 | 105 |
| 5_prime_UTR_variant | 0 | 4 | 0 | 0 | 0 | 4 | 0 | 0 | 0 | 1 | 0 |
| Inframe_Deletion | 0 | 1 | 0 | 0 | 0 | 0 | 1 | 0 | 0 | 1 | 0 |
| Missense_Variant | 0 | 7 | 6 | 9 | 1 | 1 | 4 | 0 | 0 | 10 | 8 |
| Splice_Polypyrimidine_Tract_Variant&Intron_Variant | 0 | 6 | 0 | 0 | 0 | 0 | 0 | 0 | 0 | 1 | 0 |
| Splice_region_variant&intron_variant | 0 | 1 | 0 | 0 | 0 | 0 | 0 | 0 | 0 | 1 | 0 |
| Splice_Region_Variant&Splice_Polypyrimidine_Tract_Variant&Intron_Variant | 0 | 4 | 0 | 0 | 0 | 0 | 0 | 0 | 0 | 0 | 0 |
| Synonymous_Variant | 0 | 12 | 4 | 4 | 1 | 0 | 5 | 0 | 0 | 8 | 10 |
| Upstream_Gene_Variant | 0 | 0 | 0 | 0 | 0 | 48 | 0 | 0 | 48 | 5 | 0 |
| Splice_Polypyrimidine_Tract_Variant&Splice_Region_Variant&Intron_Variant | 0 | 0 | 1 | 0 | 0 | 0 | 1 | 0 | 0 | 0 | 0 |
| 3_prime_UTR_Variant | 0 | 0 | 0 | 1 | 0 | 0 | 4 | 0 | 0 | 14 | 7 |
| Downstream_Gene_Variant | 0 | 0 | 0 | 1 | 0 | 0 | 0 | 46 | 0 | 0 | 0 |
| Splice_Region_Variant&5_prime_UTR_variant | 0 | 0 | 0 | 1 | 0 | 0 | 0 | 0 | 0 | 0 | 0 |
| Frameshift_Variant | 0 | 0 | 0 | 0 | 0 | 0 | 0 | 0 | 0 | 0 | 0 |
| Frameshift_variant&start_lost | 0 | 0 | 0 | 0 | 0 | 0 | 0 | 0 | 0 | 0 | 0 |
| Stop_gained | 0 | 0 | 0 | 0 | 0 | 1 | 0 | 0 | 0 | 0 | 0 |
| Stop_Lost | 0 | 0 | 0 | 0 | 0 | 0 | 0 | 0 | 0 | 0 | 0 |
| Splice_Donor_5th_Base_Variant&Intron_Variant | 0 | 0 | 0 | 0 | 0 | 0 | 0 | 0 | 0 | 0 | 0 |
| Splice_donor_variant | 0 | 0 | 0 | 0 | 0 | 0 | 0 | 0 | 0 | 0 | 0 |
| Start_lost | 0 | 0 | 0 | 0 | 0 | 0 | 0 | 0 | 0 | 0 | 0 |
| Intron_Variant&Non_Coding_Transcript_Variant | 0 | 0 | 0 | 0 | 0 | 0 | 0 | 0 | 0 | 0 | 0 |
| Splice_Region_Variant&Synonymous_Variant | 0 | 0 | 0 | 0 | 0 | 0 | 0 | 0 | 0 | 0 | 1 |
| Non_Coding_Transcript_Exon_Variant | 0 | 0 | 0 | 0 | 0 | 0 | 0 | 0 | 0 | 0 | 0 |
| Splice_Polypyrimidine_Tract_Variant&Intron_Variant&Non_Coding_Transcript_Variant | 0 | 0 | 0 | 0 | 0 | 0 | 0 | 0 | 0 | 0 | 0 |
| 3_prime_UTR_variant&NMD_transcript_variant | 0 | 0 | 0 | 0 | 0 | 0 | 0 | 2 | 0 | 0 | 0 |
| intron_variant&NMD_transcript_variant | 0 | 0 | 0 | 0 | 0 | 0 | 0 | 71 | 0 | 0 | 0 |
| missense_variant&NMD_transcript_variant | 0 | 0 | 0 | 0 | 0 | 0 | 0 | 4 | 0 | 0 | 0 |
| Splice_Donor_5th_Base_Variant&Intron_Variant&NMD_Transcript_Variant | 0 | 0 | 0 | 0 | 0 | 0 | 0 | 1 | 0 | 0 | 0 |
| Inframe_Insertion | 0 | 0 | 0 | 0 | 0 | 0 | 0 | 0 | 0 | 2 | 0 |
| MODIFIER | 110 | 266 | 77 | 27 | 84 | 120 | 217 | 119 | 48 | 238 | 112 |
| LOW | 0 | 23 | 5 | 5 | 1 | 0 | 6 | 1 | 0 | 10 | 11 |
| MODERATE | 0 | 8 | 6 | 9 | 1 | 1 | 5 | 4 | 0 | 13 | 8 |
| HIGH | 0 | 0 | 0 | 0 | 0 | 1 | 0 | 0 | 0 | 0 | 0 |
| SNV | 100 | 256 | 80 | 36 | 80 | 105 | 198 | 111 | 43 | 220 | 107 |
| Deletion | 7 | 22 | 3 | 4 | 4 | 10 | 15 | 8 | 3 | 24 | 15 |
| Insertion | 1 | 13 | 3 | 1 | 1 | 3 | 10 | 4 | 2 | 15 | 4 |
| Substitution | 2 | 3 | 2 | 0 | 1 | 2 | 4 | 1 | 0 | 1 | 4 |
| Indel | 0 | 3 | 0 | 0 | 0 | 2 | 1 | 0 | 0 | 1 | 1 |

**Table S5.** Accumulated effects of four known modifier variants (rs4671393 in *BCL11A,* rs7776054 in *MYB-HBS1L* intergenic regions, rs7482144 (Xmn1) and *KLF1* mutations) on the expression levels of HbF (a) and the distribution of thalassemia major and thalassemia intermedia in the β-thalassemia patient cohort (b).

1. Accumulated effects of four known modifier variants on the expression levels of HbF in the β-thalassemia patient cohort.

| Number of variants | Total number of patients | β^0^/β^0^ (n =713) | | P | β^0^/β^+^ (n=292) | | P | β^+^/β^+^(n=11) | | P |
| --- | --- | --- | --- | --- | --- | --- | --- | --- | --- | --- |
|  |  | HbF in TI (g/L,  Mean±SD) | HbF in TM (g/L, Mean±SD) |  | HbF in TI (g/L,  Mean±SD) | HbF in TM (g/L, Mean±SD) |  | HbF in TI (g/L,  Mean±SD) | HbF in TM (g/L, Mean±SD) |  |
| 0 | 33 | 8.63 (5.28) | 3.51 (2.24) | <0.001 | 13.26 (3.51) | 5.43 (8.65) | <0.001 | 0 | 0 | 0.012 |
| 1 | 556 | 14.43 (17.99) | 3.93 (5.33) |  | 17.03 (16.12) | 5.14 (6.24) |  | 10.23 (5.66) | 5.48 (1.36) |  |
| 2 | 378 | 18.87 (16.65) | 4.95 (9.51) |  | 16.70 (14.89) | 7.40 (8.54) |  | 12.86 (1.32) | 28.13 (NA) |  |
| 3 | 48 | 26.66 (26.79) | 11.80 (21.89) |  | 30.88 (18.82) | 35.95 (27.93) |  | - | - |  |
| 4 | 1 | 118.87 (NA) | - |  | - | - |  | - | - |  |

1. Accumulated effects of four known modifier variants on the distribution of thalassemia major and thalassemia intermedia in the β-thalassemia patient cohort.

| Number of variants | Total number of patients | β^0^/β^0^ (n =713) | | P | β^0^/β^+^ (n=292) | | P | β^+^/β^+^(n=11) | | P |
| --- | --- | --- | --- | --- | --- | --- | --- | --- | --- | --- |
|  |  | % of patients in TI | % of patients in TM |  | % of patients in TI | % of patients in TM |  | % of patients in TI | % of patients in TM |  |
| 0 | 30 | 2 (9.1) | 20 (90.9) | <0.001 | 3 (37.5) | 5 (62.5) | <0.001 | 0 | 0 | 0.853 |
| 1 | 472 | 17 (5.3) | 302 (94.7) |  | 38 (25.8) | 109 (74.2) |  | 5 (83.3) | 1 (16.7) |  |
| 2 | 403 | 35 (12.1) | 254 (87.9) |  | 53 (48.6) | 56 (51.4) |  | 3 (60.0) | 2 (40.0) |  |
| 3 | 101 | 30 (38.9) | 47 (61.1) |  | 13 (54.2) | 11 (45.8) |  | 0 (0.0) | 0 (0.0) |  |
| 4 | 10 | 2 (33.3) | 4 (66.7) |  | 4 (100) | 0 (0.0) |  | 0 (0.0) | 0 (0.0) |  |

**Figure S9.** The co-existence between the variants in *HBG1/2* from Hap_s1 and the disease-causing mutations in *HBB*. The composition ratios of Hap_s1, Hap_s2, and Hap_s3 among the patients with β^0^/β^0^, β^0^/β^+^, β^+^/β^+^, respectively.


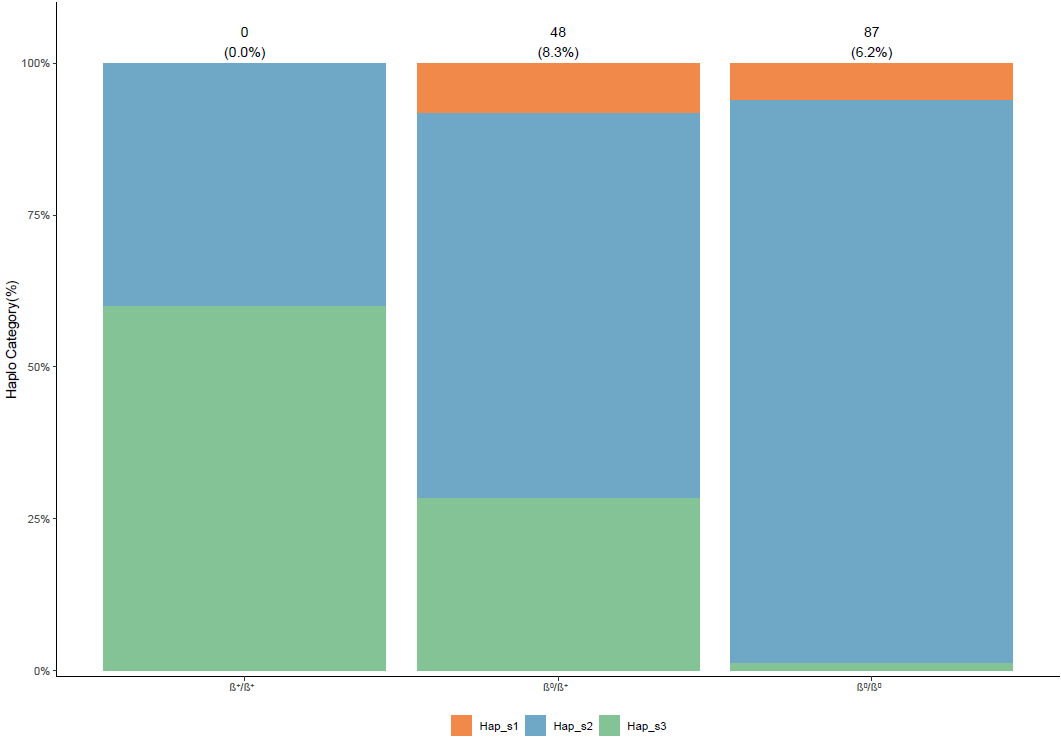


**Figure S10.** The general haplotypes of *HBA1/HBA2* regions and their phenotypic effects in the cohort of 1020 β-thalassemia patients. (A) The diagram of seven fragments in the α-globin gene cluster designed for long-read sequencing in the T-LRS panel. Among them, F2 covering the regions of *HBA1* and *HBA2* was highlighted by dotted red lines to further show the unique haplotypes of the cohort in this fragment; (B) A heatmap displaying the haplotypes of the *HBA1/HBA2* haplotypes identified from 1020 β-thalassemia patients. A total of 223 SNVs were identified from the 1020 β-thalassemia patients in this region. Each row represents one haplotype while the 223 grids in one row marked in either blue or red, denote the allele information in corresponding position. The blue color stands for a reference allele in this locus while the red stands for alteration allele. A total of 308 unique haplotypes were identified and these haplotypes were clustered into 5 main groups as shown in this figure; (C) The overview of the tree clustering results of the redundant 2040 *HBA1/HBA2* haplotypes in the patient population; (D) The LD block showing the linkage disequilibrium of the variants within the *HBA1/HBA2* genomic regions. The deeper color in each cell indicated higher R^2^ values, which means higher linkage extent between the two variants of interest; (E-G) The effects of the 5 major haplotypes in the β-thalassemia patients on the expression levels of HbF (E), the transfusion-free survival time (F) and the levels of serum ferritin (G);


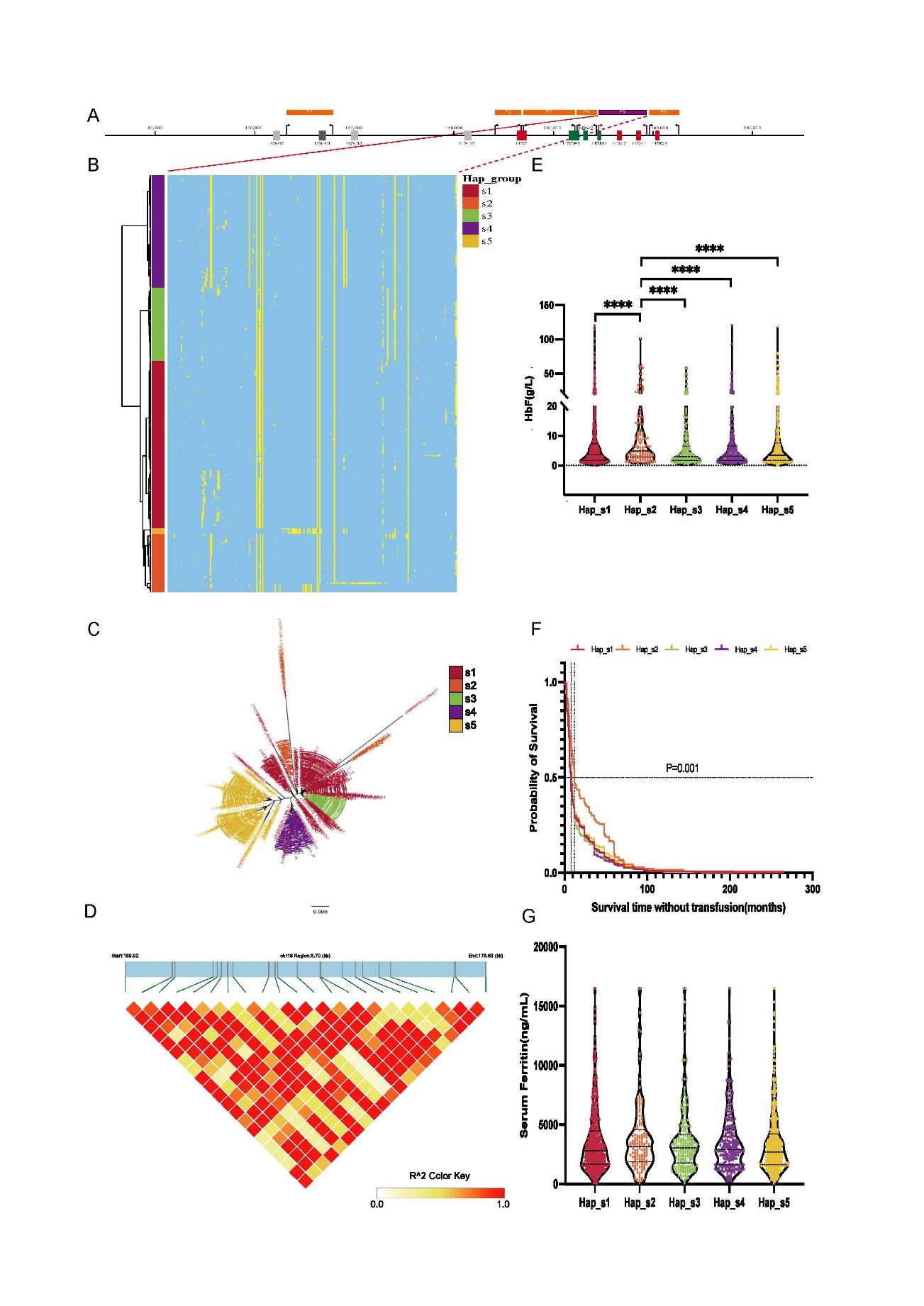


**Figure S11.** The components of α-thalassemia mutations linked with the 5 major groups of *HBA1/HBA2* haplotypes in the 1020 β-thalassemia patients.


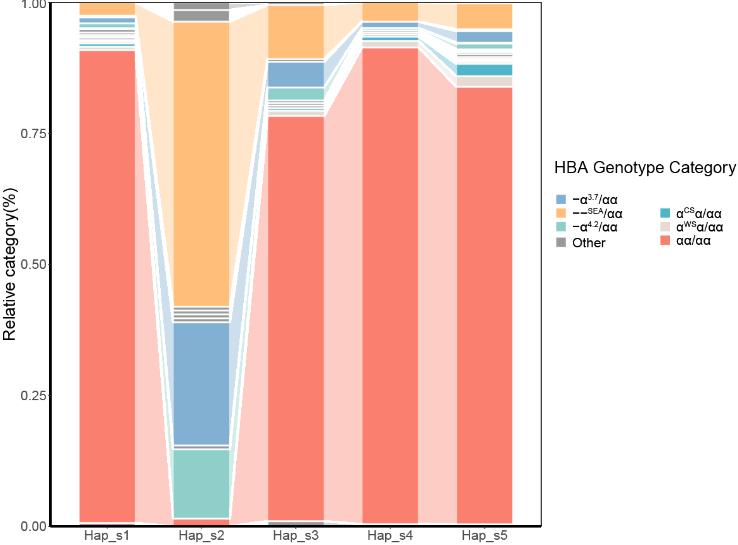

Supplement: Supplementary file 1 — Supporting Information [file ADVS-12-2410992-s001.docx]
